# Supplementary material for: Methotrexate as a corticosteroid-sparing agent in leprosy reactions: A French multicenter retrospective study
Source: PLoS Negl Trop Dis. 2023 Apr 20;17(4):e0011238. doi: 10.1371/journal.pntd.0011238 (PMC10153744; doi:10.1371/journal.pntd.0011238)
Supplement: Attachment — Submitted filename: Reviewercomments.pdf [file pntd.0011238.s001.pdf]

PLOS Neglected Tropical Diseases

Methotrexate as a corticosteroid-sparing agent in leprosy reactions: a French multicenter retrospective study

--Manuscript Draft--

**Manuscript Number:**	PNTD-D-22-01264
**Full Title:**	Methotrexate as a corticosteroid-sparing agent in leprosy reactions: a French multicenter retrospective study
**Short Title:**	Methotrexate as a corticosteroid-sparing agent in leprosy reactions
**Article Type:**	Research Article
**Keywords:**	Leprosy<br>Leprosy reaction<br>Methotrexate<br>Corticosteroid-sparing
**Abstract:**	Introduction<br>Leprosy reactions (LRs) are inflammatory responses observed in 30% to 50% of people with leprosy. First-line treatment are glucocorticoids (GCs), often administered at high doses with prolonged courses, which leads to high morbi-mortality.<br>Methotrexate (MTX) is an immunomodulating agent used to treat inflammatory diseases and has a good safety profile and worldwide availability. In this study, we describe the efficacy, GCs-sparing effect and safety of MTX in LRs.<br>Methods<br>We conducted a retrospective multicentric study in France including leprosy patients receiving MTX for a reversal reaction (RR) and/or erythema nodosum leprosum (ENL) since 2016. The primary endpoint was the rate of good response (GR) defined as the complete disappearance of inflammatory cutaneous or neurological symptoms without recurrence during MTX treatment. The secondary endpoint was the GCs-sparing effect, safety and clinical relapse after MTX discontinuation.<br>Results<br>Our study included 13 patients with LRs (8 men): 6 had ENL, 4 had RR, and 3 had both. All patients had had at least one previous course of GCs and 2 previous treatment lines before MTX.<br>Overall, 8/13 (61.5%) patients had GR, allowing for GCs-sparing and even GCs withdrawal in 6/11 (54.5%). No severe adverse effects were noted. Relapse after MTX discontinuation was frequent (42%): the median relapse time was 5.5 months (range 3-14) after stopping treatment.<br>Conclusion<br>MTX seems to be an effective alternative treatment in LRs, allowing for GCs-sparing with a good safety profile. Early introduction during LRs may lead to a better therapeutic response.<br>However, its efficacy seems to suggest prolonged therapy to avoid recurrence.
**Additional Information:**	
**Question**	**Response**
**Government Employee**<br><br>Are you or any of the contributing authors an employee of the United States government?<br><br>Manuscripts authored by one or more US Government employees are not copyrighted, but are licensed under a CC0 Public Domain Dedication, which allows	No - No authors are employees of the U.S. government.

unlimited distribution and reuse of the article for any lawful purpose. This is a legal requirement for US Government employees.<br><br>This will be typeset if the manuscript is accepted for publication.	
**Financial Disclosure**<br><br>Enter a financial disclosure statement that describes the sources of funding for the work included in this submission and the role the funder(s) played. This includes grants and any commercial funding of the work or authors.<br><br>This statement will be typeset if the manuscript is accepted for publication.<br><br>Review the submission guidelines and the instructions link below for detailed requirements and guidance.	No funding
**Competing Interests**<br><br>On behalf of all authors, disclose any competing interests that could be perceived to bias this work.<br><br>This statement will be typeset if the manuscript is accepted for publication.<br><br>Review the instructions link below and PLOS NTDs' competing interests policy to determine what information must be disclosed at submission.	None declared
**Data Availability**<br><br>Provide a **Data Availability Statement** in the box below. This statement should detail where the data used in this submission can be accessed. This statement will be typeset if the manuscript is accepted for publication.	The date that support the findings of this study are available on request from the corresponding author. The data are not publicly available due to the privacy or ethical restrictions

Before publication, authors are required to make all data underlying their findings fully available, without restriction. Review our PLOS Data Policy page for detailed information on this policy. Instructions for writing your Data Availability statement can be accessed via the Instructions link below.	

Manuscript Click here to access/download;Manuscript;Manuscrit.pdf

Methotrexate as a corticosteroid-sparing agent in leprosy reactions: a French multicenter retrospective study

Authors:

Léa Jaume[* 1], Estelle Hau[1], Gentiane Monsel[2], Antoine Mahé[3], Antoine Bertolotti[4], Antoine Petit[1], Marie Chauveau[5], Elisabeth Duhamel[6], Thierry Maisonobe [7], Martine Bagot[1], Jean-David Bouaziz[1], Faïza Mougari[8], Emmanuelle Cambau[8], Marie Jachiet[* 1]

Groupe d'infectiologie en dermatologie et des infections sexuellement transmissibles (GrIDIST)

[1]Dermatologie et vénérologie, Hôpital Saint-Louis, Université Paris Cité, Paris, France

[2]Maladies infectieuses et tropicales, Hôpital Pitié-Salpêtrière, Paris, France

[3]Dermatologie et vénérologie, CH Colmar, Colmar, France

[4]Dermatologie et vénérologie, CHU de la Réunion, Saint-Pierre, France

[5] Médecine interne, CH Saint Nazaire, Saint-Nazaire, France

[6] Medecine interne CH Saint Brieuc, Saint- Brieuc, France

[7] Neurophysiologie clinique, Pitié-Salpétrière, Paris, France

[8] Service de Mycobactériologie spécialisée et de référence, CNR des mycobactéries (laboratoire associé), APHP GHU Nord; Université Paris Cité, INSERM IAME UMR 1137 75018-Paris, France

***Corresponding authors:**

Lea.jaume@aphp.fr (LJ)

Marie.jachiet@aphp.fr (MJ)

Abstract

Introduction

Leprosy reactions (LRs) are inflammatory responses observed in 30% to 50% of people with leprosy. First-line treatment are glucocorticoids (GCs), often administered at high doses with prolonged courses, which leads to high morbi-mortality. Methotrexate (MTX) is an immunomodulating agent used to treat inflammatory diseases and has a good safety profile and worldwide availability. In this study, we describe the efficacy, GCs-sparing effect and safety of MTX in LRs.

Methods

We conducted a retrospective multicentric study in France including leprosy patients receiving MTX for a reversal reaction (RR) and/or erythema nodosum leprosum (ENL) since 2016. The primary endpoint was the rate of good response (GR) defined as the complete disappearance of inflammatory cutaneous or neurological symptoms without recurrence during MTX treatment**.** The secondary endpoint was the GCs-sparing effect, safety and clinical relapse after MTX discontinuation.

Results

Our study included 13 patients with LRs (8 men): 6 had ENL, 4 had RR, and 3 had both. All patients had had at least one previous course of GCs and 2 previous treatment lines before MTX. Overall, 8/13 (61.5%) patients had GR, allowing for GCs-sparing and even GCs withdrawal in 6/11 (54.5%). No severe adverse effects were noted. Relapse after MTX discontinuation was frequent (42%): the median relapse time was 5.5 months (range 3-14) after stopping treatment.

Conclusion: MTX seems to be an effective alternative treatment in LRs, allowing for GCs-sparing with a good safety profile. Early introduction during LRs may lead to a better therapeutic response. However, its efficacy seems to suggest prolonged therapy to avoid recurrence.

Authors summary

Leprosy reactions (LRs) are acute inflammatory episodes that complicate 30% to 50% of *Mycobacterium leprae* infections. Type 1 is also known as reversal reaction (RR), and type 2 is also known as erythema nodosum leprosum (ENL). The 2 types have relatively distinct pathogenesis and clinical features but can both be severe and progress to irreversible nerve damage and deformities. Beyond leprosy infection, the occurrence of LR is associated with neurological burden, poor quality of life, and risk of further descent into poverty for often young patients. Despite effective standardized treatment of leprosy infection with multidrug therapy (MDT) since 1981, LRs treatment is challenging. First-line treatment is primarily based on prolonged systemic glucocorticoids (GCs) therapy. The current challenge in leprosy is now to develop and evaluate new therapeutic alternatives to reduce the sequelae and morbidity of LR but also prolonged side effects of GCs. Methotrexate (MTX) is an immunomodulating agent used for treating inflammatory diseases and has a good safety profile and worldwide availability. Here, we describe the efficacy, GCs-sparing effect and safety of MTX in LR.

Introduction

Leprosy is an infectious disease caused by a slow-growing, obligate intracellular bacteria characterized as an acid-fast bacillus of the *Mycobacterium leprae* complex, including *M. lepra*e and *M. lepromatosis* [1].

Leprosy reactions (LRs) result from the host immune response to *M. leprae* complex infection and may concern 30% to 50% of all leprosy patients[2–4]. There are 2 types of LRs that can both be severe and progress to irreversible nerve damage and deformities. The type 1 reaction or reversal reaction (RR) reflects sudden restoration of cell-mediated immunity, leading to an abrupt shift of the inflammatory reaction (from T helper 2 [Th2] cells to Th1 cells) toward the tuberculoid pole, which is at greater risk of tissue damage because of compressive granulomatous formation[5]. Clinically, it is an acute and painful infiltration of pre-existing skin lesions with potentially severe and irreversible nerve damage. RR is the primary cause of neurological burden in leprosy; it may be a presenting feature of leprosy but frequently occurs secondarily after anti-bacillary treatment: the treatment-induced release of *M. leprae* antigens may restore cell-mediated immunity (Th1 cells) and explain the preferential occurrence of this phenomenon after initiating anti-bacillar therapy[6].

The type 2 reaction, or erythema nodosum leprosum (ENL), is related to a cutaneous and systemic vasculitis linked with immune complex deposits. It occurs preferentially in lepromatous forms because of the circulation of multiple *M. leprae* antigens. Clinically, it results in painful inflammatory nodules associated with systemic symptoms such as fever, arthritis, iritis, neuritis, and lymphadenitis. LRs are frequently associated with poor quality of life, severe economic burden and risk of further descent into poverty [7].

Despite effective standardized treatment for leprosy infection with antibiotic combination (i.e., MDT) since 1981, LRs treatment remains challenging. First-line treatment is primarily based on prolonged systemic glucocorticoids (GCs) therapy. The WHO recommends the introduction of 40 mg/day prednisone gradually decreased over 20 weeks [8], but the best corticosteroids regimen both in terms of dose and duration is controversial[9]. Indeed, prolonged GCs is associated with major morbidity, sometimes even death[10]. The current challenge in leprosy is to develop and evaluate new therapeutic alternatives to reduce the sequelae and morbidity of LR.

Second-line drugs such as azathioprine and ciclosporin are considered alternatives in GCs-resistant RR, but data on their efficacy and safety are lacking in this indication[11,12]. Thalidomide is an effective treatment in ENL for GCs dependence or resistance, but its multiple adverse effects including sedation, peripheral neuropathy, venous thrombosis and teratogenic effects limit its use in many endemic countries[13,14].

A high-dose regimen of clofazimine might be effective in mitigating chronic and recurrent ENL but has no effect on acute episodes[15,16]. Therapeutic alternatives such as methotrexate (MTX) pentoxifylline, phosphodiesterase-4 inhibitors and tumor necrosis factor-alpha (TNFα) inhibitors have been reported as second-line treatments in ENL in small-scale studies [17,18].

MTX is an immunomodulating agent used to treat malignant and inflammatory diseases[19,20]. MTX efficacy was described in 30 LRs cases in the literature, in 7 observations[21–28]. MTX is a cheap molecule, is widely available in many countries, has a good tolerance profile and is included in the WHO model list of essential medicines[29].

Here, we describe the efficacy, GCs-sparing effect and safety of MTX for treating LRs.

Methods

Study population and clinical data

We conducted a French multicentric retrospective study in 2 departments of dermatology (Saint-Louis hospital, Pitié-Salpêtrière hospital) and 3 departments of internal medicine-infectious disease (La Reunion, St-Brieuc and Saint-Nazaire hospitals). We included patients followed for leprosy who received MTX for RR or ENL since 2016. For each patient, the following data were collected retrospectively: age at diagnosis of leprosy, sex, country of origin, type of leprosy according to the Ridley-Jopling classification, anti-bacillary treatments received, first episode of LR, type of LR (RR or ENL), and characteristics of clinical manifestations including cutaneous and neurological. We also collected the LRs treatment before MTX, ongoing treatment at the time of MTX initiation, timing and route of administration of MTX, reduction or cessation of concomitant treatments including GCs, relapse during and after MTX discontinuation, follow-up treatments after relapse or ineffectiveness, and adverse effects during MTX treatment.

Efficacy assessment

Clinical response to MTX was assessed as good response (GR), defined as achieving complete regression of inflammatory symptoms (skin, neurological and/or systemic) and no relapse during MTX therapy. Partial response (PR) was defined as not achieving complete resolution of inflammatory symptoms or showing relapse during MTX therapy, requiring another add-on treatment to MTX or resulting in GCs dependence under MTX. Non-response (NR) was defined as persistent symptoms and/or relapse during MTX treatment, requiring MTX discontinuation or a switch to a therapeutic alternative. The primary endpoint was the GR rate. Secondary endpoints

were the GCs-sparing effect, relapse during treatment and after MTX discontinuation in GR and PR, and the MTX tolerance profile.

Data are presented as median (range) for quantitative variables and frequency (percentage) for categorical variables.

Ethics

This study was conducted in compliance with the Good Clinical Practice protocol and the Declaration of Helsinki principles. The study was approved by the local ethics committee, which waived the requirement for informed consent.

Results

Patient characteristics (Table 1)

We included 13 patients (8 men). Median age at leprosy diagnosis was 29 years (range 12-63). According to the Ridley-Jopling classification, 9 patients had lepromatous leprosy (LL), 3 borderline lepromatous (BL) leprosy and 1 borderline tuberculoid (BT) leprosy. All received MDT daily for a median of 3.5 years (range 1.12-7.5). The LRs types were ENL 46% (n = 6), RR 31% (n = 4) and both ENL and RR 23% (n=3[illegible]

All patients with ENL (n=9) had a cutaneous presentation such as painful nodules (n=5), Lucio type vasculitis (n=1), and peripheral oedema (n=4). Extra-cutaneous symptoms were uveitis (n=2), fever (n=3), polyarthritis (n=2), macrophage activation syndrom[illegible]=2), and multi-neuritis (n=1). Patients with RR (n=7) mostly had cutaneous and neurological manifestations (n=6) and one had a pure neurologic form (n=1). Two of these also had uveitis and one had polyarthritis.

Previous treatment

The median number of treatment lines received before starting MTX was 2 (range 1-4). All patients had received at least one course of GCs. The other previous treatments were thalidomide (n=9, 69%), pentoxifylline (n=4, 31%), and colchicine (n=2, 15%).

MTX

MTX initiation

MTX was prescribed subcutaneously in 7 (69%) cases, at a median dose of 20 mg/week (range 12-25) corresponding to 0.29 mg/kg body weight (0.18-0.52), for a median of 14 months (range 5-50). Eleven (85%) patients had concomitant GCs at MTX initiation, 4 (30%) had concomitant thalidomide, and 1 (8%) pentoxifylline. Time to MTX introduction (median time from first reaction to MTX introduction) was 23.5 months (range 1.6-131.).

MTX efficacy

Eight (62%) patients showed GR: 4 with ENL, 2 RR, and 2 both. They achieved complete regression of inflammatory symptoms (skin, neurological and systemic) without relapse during MTX treatment.

Two patients (15%), one with both RR and ENL *(patient 7)* and one with ENL *(patient 13)*, showed PR because they did not achieve complete disappearance of symptoms or presented relapse during MTX treatment, requiring another add-on treatment to MTX or resulting in GCs dependence under MTX.

Three patients (23%), one with ENL and 2 with RR, showed N[illegible]ecause of persistent symptoms and/or relapse during MTX treatment, requiring discontinuation of MTX or a switch to another alternative therapeutic *(patients 8, 10 and 11)*.

MTX discontinuation

Nine patients stopped MTX; 7 had response (6 GR and 1 PR), and 2 had NR. In the response group (GR and PR), MTX was stopped because of side effects despite efficacy in 3 patients, and in 4 patients who wanted to become pregnant after a prolonged remission.

The median follow-up after MTX discontinuation was 17.5 months (range 3-25).

Among the 8 patients with GR and the 2 with PR, 7 completed their treatment and 3 are still under MTX treatment. Among the 7 patients who stopped MTX after good or partial response, 3 (42%) showed relapse after a median of 5.5 months (range 3-14) (*patients 2,4 and 9)*. For all these patients, we did not observe any relapse after the therapeutic switch.

Patient characteristics are summarized in **Tables 1 and 2 and Figure 1**.

<u>Figure 1: Flow chart of patients in the study</u>

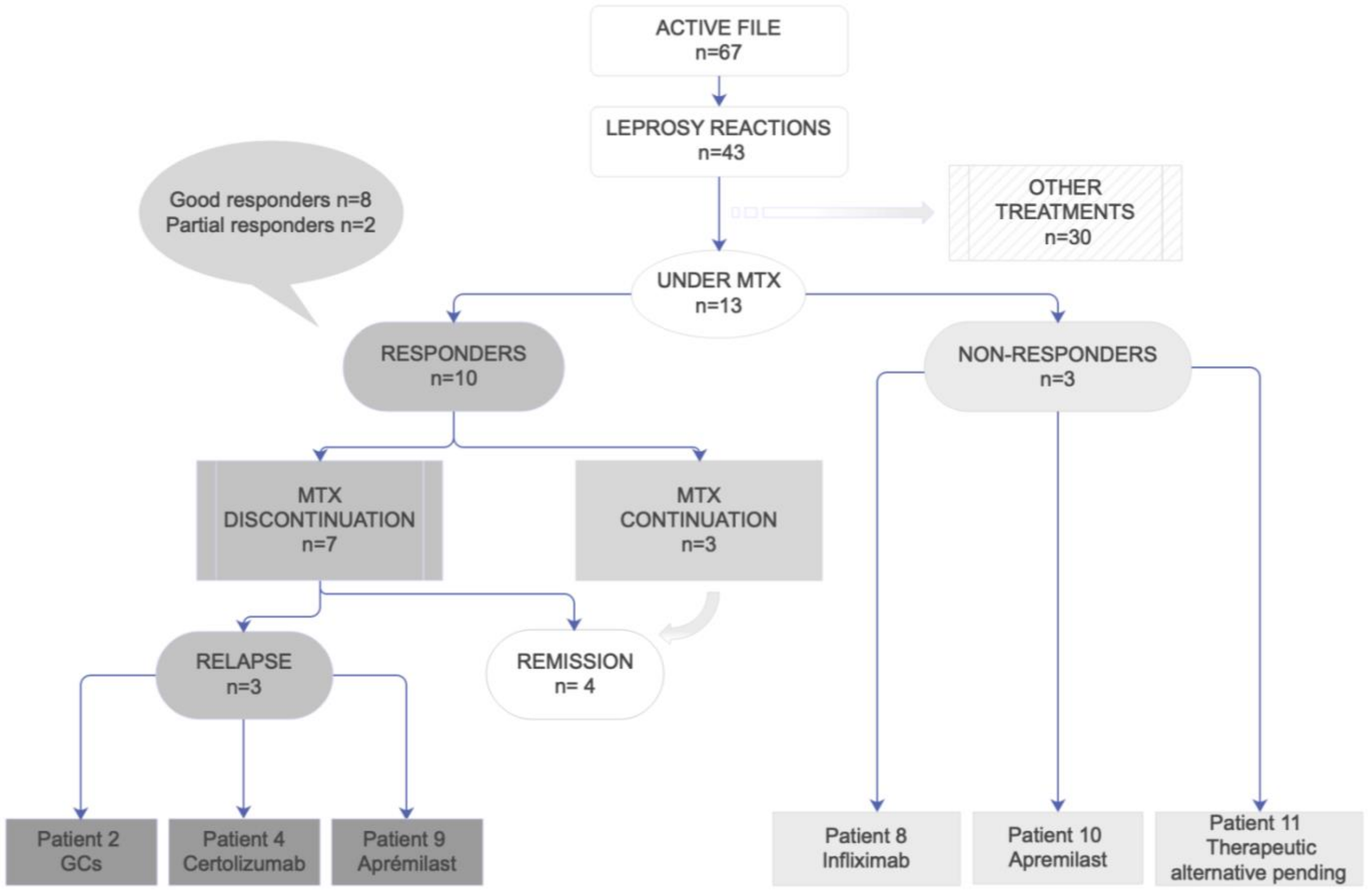

GCs-sparing effect

Six of 11 (54.5%) patients who were under GCs at MTX introduction achieved GCs withdrawal. The median time to complete withdrawal was 6 months (range 3-42). Four patients had GR, 1 PR and 1 NR.

In responders (GR and PR), the mean dose of GCs at MTX initiation was 35 mg/day (range 0-70), and was reduced to a mean of 1.5 mg/day (0-5) at MTX discontinuation or at the last follow-up for patients who were still on MTX, corresponding to a mean dose reduction of 86% (50-100%).

Sub-group analysis

We further analysed our data by identifying 2 sub-groups with predictors of GR to MTX: the first subgroup included patients with GR (n=8) and the second those with PR and NR (n=5). For both groups, we focused on the type of leprosy and LRs, the dosing regimen, the median dose, time to introduction of MTX and the number of previous treatments received. Two main predictive factors emerged from this analysis although the small number of patients did not allow for statistical analysis. First, in the GR subgroup, MTX was introduced earlier, at a median of 22 months (range 1.5-119 months) as compared with 54 months (17-131 months) in the PR and NR subgroup. Second, the administration mode in the GR group was mainly subcutaneous (n=7/8) versus oral (n=1/8), whereas in the PR and NR subgroup, the administration mode was predominantly oral (n=4/5, not reported in one case). The number of previous treatments received did not differ between the groups, with a median of 2 previous treatments for both groups and a median dose of 0.25 mg/kg (0.20-0.35) in the GR group versus 0.27 mg/kg (0.18-0.32) in the PR and NR group.

Safety

The MTX tolerance profile was acceptable. In one patient, grade II cytolysis developed in a context of hepatitis B virus chronic co-infection. One patient had gastrointestinal disorders such as nausea requiring MTX discontinuation and one had severe COVID-19; this patient had several comorbidities such as severe hypertension with hypertensive nephropathy and cardiopathy.

Table 1: Characteristics of patients with leprosy (n=13)

Female / male	5/8
Age at diagnosis (years), median (range)	29 (12-63)
Multibacillary leprosy	100%
ENL	6 (41%)
RR	4 (31%)
ENL + RR	3 (23%)
Number of therapeutic lines before MTX, median (range)	2 (1-4)
Corticosteroids	100%
Thalidomide	9/13 (69%)
Pentoxifylline	4/13 (31%)
Colchicine	2/13 (15%)
**MTX**	
Median time between 1st episode of reaction and MTX introduction (days)	714 (48-3987)
Total (months)	23 (1.6-131)
Good responders	22 (1.6-119)
Partial + non-responders	54 (17-131)
Median dose of MTX (mg/wk)	20 (12-25)
(mg/kg/body weight/week)	0.29 (0.18-0.32)
Median duration of MTX (months)	14 (5-44)
MTX administration mode	
Oral	4/11 (36%)
Subcutaneous	7/11 (64%)
Not reported	2/13
Leprosy reaction on MTX	3/13 (23%)
RR	1/13 (7%)
ENL	2/13 (15%)
**GCs THERAPY**	
GCs at MTX initiation	11 (85%)
GCs weaning under MTX	6/11 (55%)
Duration of weaning GCs (months), median (range)	6 (3-42)
Reduction of GCs	
Number of patients	11/11 (100%)
Reduction, mean (range)	-86% (50-100%)
**EVOLUTION AFTER STOPPING MTX**	
Follow-up after MTX discontinuation	13/13 (100%)
Follow-up after stopping MTX (months), median (range)	17.5 (3-25)
MTX discontinuation reason	9/13 (69%)
Ineffective	2/9 (22%)
Others	7/9 (78%)
Leprosy reaction after stopping MTX in all patients	5/9 (55%)
Among good and partial responders	3/7 (42%)
RR	1/3
ENL	2/3
RR+ ENL	0/3
Time to relapse after stopping MTX (months), median (range)	5.5 (3-14)

ENL: erythema nodosum leprum, MTX: methotrexate, RR: reverse reaction, mg/kg: milligrams per kilos body weight, GCs: glucocorticoids

Table 2: Details of clinical evolution per participant

	Type of leprosy	Type of leprosy reaction	Previous treatment for LR	MTX administration	Minimun and maximum dose of MTX: mg/wk (mg/kg body weight)	MTX duration (months)	Time between 1st LR and MTX (months)	MTX efficacy	Side effects	MTX status (months)	Relapse after MTX discontinuation (months)	Treatment required after MTX failure or discontinuation
1	BT	RR skin and neurological (facial diplegia)	GCs	SC	15-20 mg (0.25-0.3)	14	32	GOOD RESPONSE GCs withdrawal at 9 months of MTX introduction	None	Ongoing (14)		
2	LL	RR skin and neurological (sensorimotor multinevritis) ENL: skin (cutaneous nodules)	GCs, thalidomide	SC	20-25 mg (0.3-0.4)	17	23.5	GOOD RESPONSE	Gastro-intestinal disorder (nausea)	Completed	Neurological RR relapse (8): worsening of sensory neuropathy at EMG, pain hypoesthesia	GCs Restarted at 30 mg prednisone daily with gradually decreased Currently at 15 mg/day
3	LL	ENL with severity criteria: edema of the extremities, fever, MAS, episcleritis, polyarthritis	GCs	SC	15 mg (0.23)	7	21.5	GOOD RESPONSE GCs withdrawal at 10 months of MTX introduction	Stopped to become pregnant	Completed	No relapse (12)	

4	LL	ENL skin (cutaneous nodules), fever and uveitis	GCs, pentoxifylline thalidomide, colchicine	SC	15 mg (0.2)	17	76.5	GOOD RESPONSE<br>No GCs<br>Thalidomide withdrawal at 17 months of MTX introduction	Stopped to become pregnant<br>Thalidomide neuropathy	Completed	Cutaneous ENL relapse (13)	Certolizumab pegol<br>Currently at 7 months after starting treatment: no relapse to date
5	BL	ENL with severity criteria: skin (edema of the extremities, cutaneous nodules), fever, MAS and pericarditis	GCs, thalidomide	Oral	15-20 mg (0.25-0.3)	33	1.6	GOOD RESPONSE<br>GCs withdrawal at 23 months of MTX introduction<br>Thalidomide withdrawal at 8 months of MTX introduction	Stopped to become pregnant	Completed	No relapse (3)	Certolizumab pegol (MTX stop for desire of pregnancy and high risk of relapse (history of severe ENL with MAS)
6	LL	RR skin and neurological (sensorimotor multinevritis), uveitis and polyarthritis	GCs<br>Intra-occular GCs	SC	15-20 mg (0.2-0.28)	17	21.6	GOOD RESPONSE<br>No GCs<br>No relapse of uveitis at 12 months of MTX introduction	None	Ongoing (17)		
7	LL	RR skin and neurological<br>ENL "Lucio's vasculitis" type	GCs, thalidomide, pentoxifylline	Oral	17.5-25 mg (0.23-0.35)	50	58.9	PARTIAL RESPONSE<br>Adding thalidomide, and thalidomide withdrawal 1.5 year after MTX introduction<br>Pentoxifylline and GCs withdrawal 3.5 years after MTX introduction	Stopped to become pregnant	Completed	No relapse (12)	

8	LL	ENL: skin (cutaneous nodules)	GCs, colchicine, thalidomide	Oral	10-20 mg (0.22-0.45)	9,8	22.1	NON-RESPONSE Chronic ENL with skin involvement with GCs dependence at 12.5 mg prednisone	None	Ongoing (10)		Infliximab added 9 months after MTX introduction At third infliximab infusion ENL not yet controlled
9	BL	RR skin and neurological (sensorimotor multineuritis predominantly sensitive) and recurrent uveitis	GCs, thalidomide, pentoxifylline	SC	20 mg (0.25)	5	118.9	GOOD RESPONSE GCs withdrawal at 4 months of MTX introduction	Grade II cytolysis (HBV co-infection)	Completed	Uveitis relapse (4)	Intra-ocular injections of GCs and introduction of apremilast
10	LL	RR skin and neurological (radial motor mononeuropathy, sensory neuropathy)	GCs, thalidomide	ND	20-25 mg (0.23-0.3)	8	17.5	NON-RESPONSE RR with skin involvement with GCs dependence at 15 mg prednisone	None	Completed	Persistent RR skin flare-ups	Thalidomide introduction without efficacy on RR Relayed by apremilast
11	BL	RR neurological (chronic sensory neuropathy)	GCs	Oral	ND	11	131.1	NON-RESPONSE No improvement of the sensory neuropathy	None	Completed	Progressive worsening of sensory neuropathy (EMG and clinical)	The relay by pentoxifylline or an anti-TNF-α inhibitors is under discussion
12	LL	ENL skin (edema of the extremities, cutaneous nodules), polyarthritis and uveitis	GCs, thalidomide, pentoxifylline	SC	10-20 mg (0.15 0.3)	6	8.7	GOOD RESPONSE Early discontinuation of all treatments (COVID-19)	COVID-19	Completed	No relapse (16)	

13	LL	ENL skin (edema of the extremities, cutaneous nodules) and neurological (sensorimotor multinevritis)	GCs, thalidomide	Oral	10-17.5 mg (0.12-0.25)	28	42.1	PARTIAL RESPONSE<br>2 relapses: ENL skin and neurological 1 and 3 months after GCs withdrawal<br>GCs dependence at 5 mg prednisone and thalidomide	None<br>Thalidomide neuropathy	Ongoing (28)

LL: lepromatous leprosy, BL: borderline lepromatous leprosy, BT: borderline tuberculoid leprosy, LR: leprosy reaction, ENL: erythema nodosum leprum, RR: reverse reaction, SC: subcutaneous, ND: not defined, MTX: methotrexate, GCs, glucocorticoids; HBV, hepatitis B virus, EMG, electromyography, MAS, macrophagic activation syndrome, TNF, tumor necrosis factor; IV, intravenous

Discussion

This study is one of the largest cohorts reporting the benefit of MTX for treating LR (both RR and ENL), allowing for GCs-sparing. In total, 77% of patients (n=10) were considered responders (GR and PR); 62% showed complete response, defined as a total regression of inflammatory symptoms (cutaneous, neurological and systemic) and no recurrence during treatment. However, the high relapse rate, 42%, after stopping treatment argues for suspensive rather than durable efficacy, which highlights the interest of prolonged MTX treatment to maintain a clinical response, limit relapse upon discontinuation and avoid iterative courses of GCs. Moreover, MTX could be an option in refractory and/or cortico-dependent LRs and also in earlier stages, as observed in the GR subgroup in which MTX was introduced earlier. Therefore, MTX could be considered first-line therapy combined with GCs to facilitate GCs-sparing and reduce the risk of recurrence and transition to chronic LR.

We acknowledge some limitations to our study, the main one being the retrospective design associated with heterogeneous patient profiles and non-standardised treatment strategies. Our sample size was small even though it is the largest cohort in the literature.

We identified 7 published studies including 30 cases in PubMed. One was a randomized study from Bangladesh comparing the efficacy and safety of MTX plus prednisolone (n=10 patients) versus prednisolone monotherapy (n=9) during 6 months in ENL. The others were case reports [21–28] .

Our data were globally concordant with the literature, but some points need to be highlighted. First, concerning the MTX course, the dosing and administration regimen differed. Our median MTX dose was twice as high as that in the literature (20 mg/week corresponding to 0.29 mg/kg body weight vs 7.5 mg/week corresponding to 0.14 mg/kg body weight) and was mostly administered subcutaneously, whereas for all the cases in the literature, MTX was given orally.

Our therapeutic choice is based on several publications that suggest better efficacy of subcutaneous than oral treatment in inflammatory diseases such as psoriasis and rheumatoid arthritis, with a median dose of 0.3 mg/kg/week usually used in inflammatory diseases [19, 30,, 31]. Second, the GCs tapering schedule was faster in our patients than in literature case reports (median of 6 vs 12 months). This schedule may explain in part a higher relapse rate under MTX in our cohort (3/13 cases vs 2/30 in the literature), but the low rate of recurrence of LRs under MTX in the literature is also probably related to major publication bias (20 case reports, 10 cases in a randomized trial) and a shorter follow-up after MTX discontinuation (12 vs 17.5 months in our study). Finally, MTX was introduced earlier after a first episode of LR in the literature cases (median of 11 vs 23 months after an initial diagnosis of LR in our cases) but with a similar median of 2 previous treatments.

Currently, the first-line treatment of LR is based on GCs therapy. Used as pre-emptive therapy, GCs did not demonstrate clear-cut benefits. Moreover, the optimal dose, decrease pattern and length of treatment are not yet clearly established. Rao *et al.* demonstrated that a 20-week GCs regimen was better than a 12-week regimen[9], but in real life observations, GCs must be prolonged, including at high doses (up to 1 mg/kg body weight), thus increasing the risk of immunosuppression and corticosteroid-induced diabetes, bone demineralization and hypertension. However, about 40% of individuals with RR do not show clinical improvement. Especially with nerve damage, GCs seem to be ineffective [32, 33].

Azathioprine (AZA), metabolized to mercaptopurine by thiopurine methyltransferase enzyme, acts by inhibiting T-cell immunity. Its efficacy on RR has been evaluated in AZALEP, an Indian randomised double-blind study of 345 patients with leprosy receiving GCs plus AZA or placebo for RR. The trial showed that adding AZA to GCs did not decrease the clinical reaction severity score or reduce the recurrence rate of RR. Its efficacy also seems suspensive, with better outcomes for the 48-week treatment group[11].

Ciclosporin has also been tested in an Ethiopian randomized trial comparing ciclosporin and prednisone efficacy and the tolerance profile in acute RR. The 2 groups did not differ in improved clinical outcomes (skin and nerve), tolerance profile or rate of RR recurrence (85%). Moreover, patients on ciclosporin required additional prednisolone to treat recurrences.[12]

With regard to the management of ENL, the chronic course and frequency of relapses (40% of ENL) are difficult to prevent and often justify prolonged and/or repeated courses of GCs [34]. Clofazimine is widely used for treating ENL; it has no effect on acute episodes but might be effective in preventing chronic and recurrent ENL. The induced skin pigmentation is sometimes stigmatising and is one of the limiting factors in its long-term use[15, 16]. Thalidomide is an effective therapeutic option in moderate to severe ENL. An Indian retrospective study of 102 patients with ENL argues for early initiation of thalidomide to achieve faster and longer remission owing to its efficacy in preventing recurrences [13]. However, it is also an irrelevant option in nerve damage, and its use is limited by multiple adverse effects such as its teratogenicity.

Other therapeutic alternatives such as pentoxifylline and apremilast, an oral phosphodiesterase-4 inhibitor sharing a common molecular structure with thalidomide, seem to avoid recurrence and allow GCs-sparing[18, 35].

Finally, some case series concerning the value of biotherapies such as TNF-α inhibitors have recently been published. A recent review identified 4 published cases and one unpublished report of refractory ENL effectively treated with a TNF-α inhibitor (infliximab or etanercept)[17]. All patients previously received thalidomide and prednisone; 2 also received pentoxifylline and one AZA. GCs-sparing was reported in all patients under anti-TNFα, and 3 showed long-term remission after treatment discontinuation. These data are promising and must be confirmed in larger cohorts. Indeed, few therapies have demonstrated prolonged efficacy after treatment discontinuation. TNF-α inhibitors probably have a long-lasting immunological action that can

modify the evolutionary trajectory of the disease. Among our patients, 2 received certolizumab pegol following relapse after discontinuation of MTX and one received infliximab in addition to MTX. The long-term outcome was favourable for 3 of them. No adverse events were reported. Unfortunately, the use of anti-TNF-α is limited by the cost of agents and the availability in developing countries. Thus, MTX is currently the best alternative for LRs because of its GCs-sparing efficacy, safety, availability and cost.

A large randomized controlled trial is under way to assess the efficacy of MTX in conjunction with prednisolone in managing ENL, thus demonstrating the promising effectiveness of this treatment[36]. Further prospective and controlled studies are needed to demonstrate the efficacy, safety and feasibility of MTX treatment for both type 1 and 2 LR to draft new guidelines.

References

1. Maymone, M. B. C. Leprosy: Clinical aspects and diagnostic techniques. *J AM ACAD DERMATOL* **83**, 14 (2020).
2. Scollard, D. M. *et al.* The continuing challenges of leprosy. *Clin Microbiol Rev* **19**, 338–381 (2006).
3. Kamath, S., Vaccaro, S. A., Rea, T. H. & Ochoa, M. T. Recognizing and managing the immunologic reactions in leprosy. *J Am Acad Dermatol* **71**, 795–803 (2014).
4. Lockwood, D. N. J. *et al.* The histological diagnosis of leprosy type 1 reactions: identification of key variables and an analysis of the process of histological diagnosis. *J Clin Pathol* **61**, 595–600 (2008).
5. Fonseca, A. B. de L. *et al.* The influence of innate and adaptative immune responses on the differential clinical outcomes of leprosy. *Infect Dis Poverty* **6**, 5 (2017).
6. Rose, P. & Waters, M. F. Reversal reactions in leprosy and their management. *Lepr Rev* **62**, 113–121 (1991).
7. Chandler, D. J. *et al.* Household costs of leprosy reactions (ENL) in rural India. *PLoS Negl Trop Dis* **9**, e0003431 (2015).
8. World Health Organization. *Leprosy hansen disease: management of reactions and prevention of disabilities: technical guidance*. (World Health Organization, 2020).
9. Rao, P. S. S. S., Sugamaran, D. S. T., Richard, J. & Smith, W. C. S. Multi-centre, double blind, randomized trial of three steroid regimens in the treatment of type-1 reactions in leprosy. *Lepr Rev* **77**, 25–33 (2006).
10. Walker, S. L., Lebas, E., Doni, S. N., Lockwood, D. N. J. & Lambert, S. M. The mortality associated with erythema nodosum leprosum in Ethiopia: a retrospective hospital-based study. *PLoS Negl Trop Dis* **8**, e2690 (2014).
11. Lockwood, D. N. J. *et al.* AZALEP a randomized controlled trial of azathioprine to treat leprosy nerve damage and Type 1 reactions in India: Main findings. 22.
12. Lambert, S. M. *et al.* A Randomized Controlled Double Blind Trial of Ciclosporin versus Prednisolone in the Management of Leprosy Patients with New Type 1 Reaction, in Ethiopia. *PLoS Negl Trop Dis* **10**, e0004502 (2016).
13. Kaur, I., Dogra, S., Narang, T. & De, D. Comparative efficacy of thalidomide and prednisolone in the treatment of moderate to severe erythema nodosum leprosum: a randomized study. *Australas J Dermatol* **50**, 181–185 (2009).
14. Van Veen, N. H. J., Lockwood, D. N. J., Van Brakel, W. H., Ramirez, J. & Richardus, J. H. Interventions for erythema nodosum leprosum. A Cochrane review. *Lepr Rev* **80**, 355–372 (2009).
15. Balagon, M., Saunderson, P. R. & Gelber, R. H. Does clofazimine prevent erythema nodosum leprosum (ENL) in leprosy? A retrospective study, comparing the experience of multibacillary patients receiving either 12 or 24 months WHO-MDT. *Lepr Rev* **82**, 213–221 (2011).
16. Maghanoy, A., Balagon, M., Saunderson, P. & Scheelbeek, P. A prospective randomised, double-blind, placebo controlled trial on the effect of extended clofazimine on Erythema Nodosum Leprosum (ENL) in multibacillary (MB) leprosy. *Leprosy Review* **88**, 208–2016 (2017).
17. Cogen, A. L. *et al.* Biologics in Leprosy: A Systematic Review and Case Report. *Am J Trop Med Hyg* **102**, 1131–1136 (2020).
18. Narang, T., Ashraf, R., Kaushik, A. & Dogra, S. Apremilast in multibacillary leprosy patients with chronic and recurrent erythema nodosum leprosum: A prospective single centre pilot study. *J Eur Acad Dermatol Venereol* (2021) doi:10.1111/jdv.17585.

19. Swierkot, J. & Szechiński, J. Methotrexate in rheumatoid arthritis. *Pharmacol Rep* **58**, 473–492 (2006).
20. Warren, R. B. *et al.* British Association of Dermatologists' guidelines for the safe and effective prescribing of methotrexate for skin disease 2016. *British Journal of Dermatology* **175**, 23–44 (2016).
21. Biosca, G. & Casallo, S. Methotrexate Treatment for Type 1 (Reversal) Leprosy Reactions. 3.
22. Kar, B. R. & Babu, R. Methotrexate in resistant ENL. *Int J Lepr Other Mycobact Dis* **72**, 480–482 (2004).
23. Nagar, R., Khare, S. & Sengar, S. S. Effectiveness of Methotrexate in prednisolone and thalidomide resistant cases of Type 2 lepra reaction: report on three cases. *LEPROSY* **86**, 379–382 (2015).
24. Saxena, S. *et al.* Severe type 2 leprosy reaction with COVID-19 with a favourable outcome despite continued use of corticosteroids and methotrexate and a hypothesis on the possible immunological consequences. *Int J Infect Dis* **103**, 549–551 (2021).
25. Hossain, D. Management of chronic neuritis with a combination regimen of lower doses prednisolone and methotrexate: a brief report. *Leprosy Review* **87**, 118–121 (2016).
26. Hossain, D. Using methotrexate to treat patients with ENL unresponsive to steroids and clofazimine: a report on 9 patients. *Lepr Rev* **84**, 105–112 (2013).
27. Hasan, Z., Mowla, M. R., Angkur, D. M. & Khan, M. I. Efficacy and Safety of Prednisolone Monotherapy Versus Prednisolone Plus Methotrexate in Erythema Nodosum Leprosum (Type 2 Lepra Reaction). *International Journal of Dermatology and Venereology* **3**, 205–210 (2020).
28. Perez-Molina, J. A. *et al.* Use of methotrexate for leprosy reactions. Experience of a referral center and systematic review of the literature. *Travel Med Infect Dis* **37**, 101670 (2020).
29. WHO-MVP-EMP-IAU-2019.06-eng.pdf.
30. Bianchi, G., Caporali, R., Todoerti, M. & Mattana, P. Methotrexate and Rheumatoid Arthritis: Current Evidence Regarding Subcutaneous Versus Oral Routes of Administration. *Adv Ther* **33**, 369–378 (2016).
31. Attwa, E. M., Elkot, R. A., Abdelshafey, A. S. & Hafez, A. R. Subcutaneous methotrexate versus oral form for the treatment and prophylaxis of chronic plaque psoriasis. *Dermatol Ther* **32**, e13051 (2019).
32. Van Veen, N. H. J., Nicholls, P. G., Smith, W. C. S. & Richardus, J. H. Corticosteroids for treating nerve damage in leprosy. *Cochrane Database Syst Rev* CD005491 (2016) doi:10.1002/14651858.CD005491.pub3.
33. Wagenaar, I. *et al.* Effectiveness of 32 versus 20 weeks of prednisolone in leprosy patients with recent nerve function impairment: A randomized controlled trial. *PLoS Negl Trop Dis* **11**, e0005952 (2017).
34. Negera, E. *et al.* Clinico-pathological features of erythema nodosum leprosum: A case-control study at ALERT hospital, Ethiopia. *PLoS Negl Trop Dis* **11**, e0006011 (2017).
35. Sales, A. M. *et al.* Double-blind trial of the efficacy of pentoxifylline vs thalidomide for the treatment of type II reaction in leprosy. *Braz J Med Biol Res* **40**, 243–248 (2007).
36. London School of Hygiene and Tropical Medicine. *Methotrexate and Prednisolone Study in Erythema Nodosum Leprosum (MaPS in ENL*. https://clinicaltrials.gov/ct2/show/NCT03775460 (2021).
