# Supplementary material for: Methotrexate as a corticosteroid-sparing agent in leprosy reactions: A French multicenter retrospective study
Source: PLoS Negl Trop Dis. 2023 Apr 20;17(4):e0011238. doi: 10.1371/journal.pntd.0011238 (PMC10153744; doi:10.1371/journal.pntd.0011238)
Supplement: Attachment — Submitted filename: Revised Manuscript.pdf [file pntd.0011238.s004.pdf]

PLOS Neglected Tropical Diseases

# Methotrexate as a corticosteroid-sparing agent in leprosy reactions: a French multicenter retrospective study

--Manuscript Draft--

| | |
|---|---|
| **Manuscript Number:** | PNTD-D-22-01264R1 |
| **Full Title:** | Methotrexate as a corticosteroid-sparing agent in leprosy reactions: a French multicenter retrospective study |
| **Short Title:** | Methotrexate as a corticosteroid-sparing agent in leprosy reactions |
| **Article Type:** | Research Article |
| **Keywords:** | Leprosy<br>Leprosy reaction<br>Methotrexate<br>Corticosteroid-sparing |
| **Abstract:** | Introduction<br>Leprosy reactions (LRs) are inflammatory responses observed in 30%-50% of people with leprosy. First-line treatment is glucocorticoids (GCs), often administered at high doses with prolonged courses, resulting in high morbi-mortality. Methotrexate (MTX) is an immunomodulating agent used to treat inflammatory diseases and has an excellent safety profile and worldwide availability. In this study, we describe the efficacy, GCs-sparing effect and safety of MTX in LRs.<br>Methods<br>We conducted a retrospective multicentric study in France consisting of leprosy patients receiving MTX for a reversal reaction (RR) and/or erythema nodosum leprosum (ENL) since 2016. The primary endpoint was the rate of good response (GR) defined as the complete disappearance of inflammatory cutaneous or neurological symptoms without recurrence during MTX treatment. The secondary endpoint was the GCs-sparing effect, safety and clinical relapse after MTX discontinuation.<br>Results<br>Our study included 13 patients with LRs (8 men, 5 women): 6 had ENL and 7 had RR. All patients had had at least one previous course of GCs and 2 previous treatment lines before starting MTX. Overall, 8/13 (61.5%) patients had GR, allowing for GCs-sparing and even GCs withdrawal in 6/11 (54.5%). No severe adverse effects were observed. Relapse after MTX discontinuation was substantial (42%): the median relapse time was 5.5 months (range 3-14) after stopping treatment.<br>Conclusion: MTX seems to be an effective alternative treatment in LRs, allowing for GCs-sparing with a good safety profile. Furthermore, early introduction during LRs may lead to a better therapeutic response. However, its efficacy seems to suggest prolonged therapy to prevent recurrence. |
| **Additional Information:** | |
| **Question** | **Response** |
| **Government Employee**<br><br>Are you or any of the contributing authors an employee of the United States government?<br><br>Manuscripts authored by one or more US Government employees are not copyrighted, but are licensed under a CC0 Public Domain Dedication, which allows unlimited distribution and reuse of the | No - No authors are employees of the U.S. government. |

Powered by Editorial Manager® and ProduXion Manager® from Aries Systems Corporation

| | |
|---|---|
| article for any lawful purpose. This is a legal requirement for US Government employees.<br><br>This will be typeset if the manuscript is accepted for publication. | |
| **Financial Disclosure**<br><br>Enter a financial disclosure statement that describes the sources of funding for the work included in this submission and the role the funder(s) played. This includes grants and any commercial funding of the work or authors.<br><br>This statement will be typeset if the manuscript is accepted for publication.<br><br>Review the submission guidelines and the instructions link below for detailed requirements and guidance. | The authors received no specific funding for this work. |
| **Competing Interests**<br><br>On behalf of all authors, disclose any competing interests that could be perceived to bias this work.<br><br>This statement will be typeset if the manuscript is accepted for publication.<br><br>Review the instructions link below and PLOS NTDs' competing interests policy to determine what information must be disclosed at submission. | None declared |
| **Data Availability**<br><br>Provide a **Data Availability Statement** in the box below. This statement should detail where the data used in this submission can be accessed. This statement will be typeset if the manuscript is accepted for publication. | The data that support the findings of this study are available on request from Miss Douniazed Chabane, who is data manager douniazed.chabane@aphp.fr |

| | |
|---|---|
| Before publication, authors are required to make all data underlying their findings fully available, without restriction. Review our PLOS Data Policy page for detailed information on this policy. Instructions for writing your Data Availability statement can be accessed via the Instructions link below. | |

*Powered by Editorial Manager® and ProduXion Manager® from Aries Systems Corporation*

Manuscript Click here to access/download;Manuscript;Manuscript R1.docx

# Methotrexate as a corticosteroid-sparing agent in leprosy reactions: a French multicenter retrospective study

**Authors:**

Léa Jaume[* 1], Estelle Hau[1], Gentiane Monsel[2], Antoine Mahé[3], Antoine Bertolotti[4], Antoine Petit[1], Britney Le[5], Marie Chauveau[6], Elisabeth Duhamel[7], Thierry Maisonobe [8], Martine Bagot[1], Jean-David Bouaziz[1, 5], Faïza Mougari[9], Emmanuelle Cambau[9], Marie Jachiet[* 1, 5]

Groupe d'infectiologie en dermatologie et des infections sexuellement transmissibles (GrIDIST)

[1]Dermatologie et vénérologie, Hôpital Saint-Louis, Université Paris Cité, Paris, France

[2]Maladies infectieuses et tropicales, Hôpital Pitié-Salpêtrière, Paris, France

[3]Dermatologie et vénérologie, CH Colmar, Colmar, France

[4]Dermatologie et vénérologie, CHU de la Réunion, Saint-Pierre, France

[5] INSERM Human Immunology, Physiopathology and Immunotherapy U976, Hospital Saint-Louis, Paris, France

[6] Médecine interne, CH Saint Nazaire, Saint-Nazaire, France

[7] Medecine interne CH Saint Brieuc, Saint- Brieuc, France

[8] Neurophysiologie clinique, Pitié-Salpétrière, Paris, France

[9] Service de Mycobactériologie spécialisée et de référence, CNR des mycobactéries (laboratoire associé), APHP GHU Nord; Université Paris Cité, INSERM IAME UMR 1137 75018-Paris, France

***Corresponding authors:**

**Lea.jaume@aphp.fr (LJ)**

Marie.jachiet@aphp.fr (MJ)

# Abstract

**Introduction**

Leprosy reactions (LRs) are inflammatory responses observed in 30%-50% of people with leprosy. First-line treatment is glucocorticoids (GCs), often administered at high doses with prolonged courses, resulting in high morbi-mortality. Methotrexate (MTX) is an immunomodulating agent used to treat inflammatory diseases and has an excellent safety profile and worldwide availability. In this study, we describe the efficacy, GCs-sparing effect and safety of MTX in LRs.

**Methods**

We conducted a retrospective multicentric study in France consisting of leprosy patients receiving MTX for a reversal reaction (RR) and/or erythema nodosum leprosum (ENL) since 2016. The primary endpoint was the rate of good response (GR) defined as the complete disappearance of inflammatory cutaneous or neurological symptoms without recurrence during MTX treatment. The secondary endpoint was the GCs-sparing effect, safety and clinical relapse after MTX discontinuation.

**Results**

Our study included 13 patients with LRs (8 men, 5 women): 6 had ENL and 7 had RR. All patients had had at least one previous course of GCs and 2 previous treatment lines before starting MTX. Overall, 8/13 (61.5%) patients had GR, allowing for GCs-sparing and even GCs withdrawal in 6/11 (54.5%). No severe adverse effects were observed. Relapse after MTX discontinuation was substantial (42%): the median relapse time was 5.5 months (range 3-14) after stopping treatment.

**Conclusion:** MTX seems to be an effective alternative treatment in LRs, allowing for GCs-sparing with a good safety profile. Furthermore, early introduction during LRs may lead to a better

therapeutic response. However, its efficacy seems to suggest prolonged therapy to prevent recurrence.


## Authors’ summary

Leprosy reactions (LRs) are acute inflammatory episodes that complicate 30% to 50% of *Mycobacterium leprae* infections. Type 1 is known as reversal reaction (RR), and type 2 is known as erythema nodosum leprosum (ENL). The two types have relatively distinct pathogenesis and clinical features; furthermore, they can advance to become severe and progress to irreversible nerve damage and deformities. Beyond leprosy infection, LR can bring about neurological burden, poor quality of life, and risk of further descent into poverty for often young patients. Despite effective standardized treatment of leprosy infection with multidrug therapy (MDT) since 1981, treating LRs prove to be challenging. First-line treatment of LR is based on prolonged systemic glucocorticoids (GCs) therapy. The current challenge in leprosy is now to develop and evaluate new therapeutic alternatives to reduce not only the sequelae and morbidity of LR but also the lasting side effects of GCs. Thalidomide is an effective alternative in moderate to severe ENL, but its use is limited given its numerous adverse effects. Methotrexate (MTX) is an immunomodulating agent used to treat inflammatory diseases and has an excellent safety profile and worldwide availability. Here, we describe the efficacy, GCs-sparing effect, and safety of MTX in LR.

# Introduction

Leprosy is an infectious disease caused by an acid-fast bacillus of the *Mycobacterium leprae* complex, including *M. lepra*e and *M. lepromatosis* (1).

Leprosy reactions (LRs) result from the host immune response to *M. leprae* complex infection and may concern 30% to 50% of all leprosy patients (2–4). There are two types of LRs that can both evolve to become severe and progress to irreversible nerve damage and deformities. Type 1 reaction, also known as reversal reaction (RR), is a sudden restoration of cell-mediated immunity, trigerring an abrupt shift of the inflammatory reaction (from T helper 2 (Th2) cells to T helper 1 (Th1) cells) toward the tuberculoid pole; this shift causes greater risk of tissue damage due to compressive granulomatous formation (5). Clinically, RR is an acute and painful infiltration of pre-existing skin lesions with potentially severe and irreversible nerve damage. Type 2 reaction, also known as erythema nodosum leprosum (ENL), is a cutaneous and systemic vasculitis linked with immune complex deposits. Clinically, it results in painful inflammatory nodules associated with systemic symptoms such as fever, arthritis, iritis, neuritis, and lymphadenitis.

Despite effective standardized treatment for leprosy infection with antibiotic combination (i.e., MDT) since 1981, treating LRs remains challenging. First-line treatment is primarily based on prolonged systemic glucocorticoids (GCs) therapies, associated with major morbidity and sometimes, even death (6). The current challenge in leprosy is to develop and evaluate new therapeutic alternatives to reduce the sequelae and morbidity of LR (7).

Second-line drugs such as azathioprine and ciclosporin in RR and thalidomide or high-dose of clofazimine in ENL are considered alternatives in GCs-resistant LR, but their use is limited due to their adverse effects (8), (9), (10), (11), (12), (13). Therapeutic alternatives such as methotrexate

(MTX), pentoxifylline, phosphodiesterase-4 inhibitors and tumor necrosis factor-alpha (TNFα) inhibitors have been reported as second-line treatments in ENL in small-scale studies (14),(15).

MTX is an immunomodulating agent used to treat malignant and inflammatory diseases(16),(17). Its efficacy has been described in 30 LRs cases in the literature, in 7 observations (18–25). MTX is a cheap molecule, is widely available in many countries, has a good tolerance profile, and is included in the WHO model list of essential medicines (26).

This is the reason why we performed a retrospective study to describe the efficacy, GCs-sparing effect, and safety of MTX for treating LRs.

# Methods

## Study design and inclusion criteria

We conducted a multicentric retrospective study carried out after a national case call (through the GRIDIST (Infectious and Sexually Transmitted Infections Group of the French Society of Dermatology) and the national multidisciplinary leprosy meeting. All leprosy patients with type 1 or type 2 LR treated at least 3 months with MTX between June 2016 and August 2021 in France were included.

## MTX regimen and monitoring

All patients had a mandatory assessment of full blood cell count, serum transaminase levels, serum creatinine with computation of creatinine clearance, serological test for the hepatitis viruses B and C, serum albumin assay and a chest radiograph before starting MTX.

Regarding dose regimen, MTX was given at a minimal dose of 0.15 up to 0.3 mg/kg/week, as typically administered in inflammatory diseases (16).

Monitoring on MTX was consistent with current recommendations (27) (full blood cell counts, serum transaminase and creatinine assays at least one a month for the first 3 months then every 4-12 weeks). Folate supplementation was given at a minimal dosage of 5 to 10 mg once a week at a distance from MTX dose (28).

## Data collection and definitions

Clinical and biological information was obtained from digital medical records for each patient. Recorded data included date of diagnosis of leprosy, gender, country of origin, type of leprosy according to the Ridley-Jopling classification, anti-bacillary treatments received, type of LR (RR

or ENL), date of first episode of LR and characteristics of clinical manifestations including cutaneous and neurological. We also collected the LRs treatment before MTX, ongoing treatment at the time of MTX initiation, timing and route of administration of MTX, relapse on and after MTX therapy, alternative treatments after relapse, and MTX ineffectiveness.

Adverse events (AEs) were retrospectively collected and graded according to the Common Terminology Criteria for Adverse Events (CTCAE) v5.0.

As there is no standard recommendation to evaluate MTX therapeutic response, we have used clinically valuable outcomes to evaluate MTX clinical response for this study. We defined three types of clinical response to MTX. Good response (GR) was defined as achieving complete regression of inflammatory symptoms (skin, neurological and/or systemic) and having no relapse during MTX therapy. Partial response (PR) was defined as not achieving complete resolution of inflammatory symptoms or showing relapse during MTX therapy, requiring another add-on treatment to MTX or resulting in GCs dependence under MTX. Non-response (NR) was described as persistent symptoms and/or relapse during MTX treatment, requiring MTX discontinuation or a switch to a therapeutic alternative.

Data are presented as median and mean (range) for quantitative variables and frequency (percentage) for categorical variables.

## Outcomes

The primary endpoint was the GR rate. Secondary endpoints were the GCs-sparing effect, number of relapse after MTX discontinuation in GR and PR groups and MTX tolerance profile.

## Ethics

This study was conducted in compliance with the Good Clinical Practice protocol and the Declaration of Helsinki principles. The study was approved by the local ethics committee (CPP Paris île de France 4), who waived the requirement for informed consent.

# Results

## Patient characteristics (Table 1)

A total of 13 patients (8 men, 5 women) fulfilled inclusion criteria and received MTX. Median age at leprosy diagnosis was 29 years (range 12-63). The LRs types were ENL 46% (n = 6) and RR 54% (n = 7). All patients with ENL (n=6) had a cutaneous presentation such as painful nodules (n=6) and/or peripheral oedema (n=4). Extra-cutaneous symptoms were fever (n=3), polyarthritis (n=2), and neuropathy (n=1).

Patients with RR (n=7) mostly had cutaneous and neurological manifestations (n=6) and one had a pure neurologic form (n=1). Two of which had uveitis and one had polyarthritis.

## Previous treatments received for LR

Regarding leprosy, all patients were treated with MDT daily for a median of 2.3 years (range 1.12-7.5). They all received rifampicine and clofazimine. Clofazimine was continued after completion of MDT to prevent LRs and was not included in the median time of MDT treatment. Twelve (92%) patients received disulone[illegible] one of them was switched to ofloxacin due to a dapsone-induced anemia. One patient had a G6PD deficiency and was treated with clarithromycin. Aside from clofazimine, no MDT treatment was given during MTX therapy.

Regarding LRs, the median number of treatment lines received before starting MTX was 2 (range 1-4). All patients had received at least one previous course of GCs. The other previous treatments were thalidomide (n=9, 69%), pentoxifylline (n=4, 31%), and colchicine (n=2, 15%).

## Treatment continued with MTX

At the time of MTX initiation, 11 (85%) patients were taking GCs with a mean dose of 46 mg (0-90), 4 patients (31%) were under thalidomide with a mean dose of 112 mg (50-200) and one had pentoxifylline (8%).

## MTX efficacy on LR

MTX was initiated after a median time of 23 months of LR evolution (1.6-131).

MTX was prescribed subcutaneously in 7 (69%) cases, at a median dose of 20 mg/week (range 12-25) corresponding to 0.29 mg/kg body weight (0.18-0.52), for a median time of 14 months (range 5-50).

GR was observed in 8 (62%) patients. They achieved complete regression of inflammatory symptoms (skin, neurological, and systemic) without relapse during MTX treatment.

Two patients (15%), *(patient 7 and patient 13)* had a PR because they did not achieve complete disappearance of symptoms or presented relapse during MTX treatment, requiring another add-on treatment to MTX or resulting in GCs dependence under MTX.

Three patients (23%) were defined as NR due to persistent symptoms and/or relapse during MTX treatment, requiring discontinuation of MTX or a switch to another alternative therapeutic *(patients 8, 10 and 11)*.

In the GR subgroup (n=8), MTX was introduced with a median of 22 months after LR diagnosis (range 1.5-119 months) *versus* 54 months (17-131 months) in the PR and NR subgroup (n=5). The

administration mode in the GR group was mainly subcutaneous (n=7/8) *versus* oral (n=1/8), whereas in the PR and NR subgroup, the administration mode was predominantly oral (n=4/5, not reported in one case).

The number of previous treatments received did not differ between the groups, with a median number of 2 previous treatments for both groups and a median dose of MTX of 0.25 mg/kg (0.20-0.35) in the GR group *versus* 0.27 mg/kg (0.18-0.32) in the PR and NR group.

# GCs-sparing effect

Six of 11 (54.5%) patients who were under GCs at MTX introduction achieved GCs withdrawal. The median time to complete withdrawal was 6 months (range 3-42). In all patients, the mean dose of GCs at MTX initiation was 46 mg/day (range 0-90), and was reduced to a mean of 4 mg/day (0-20) at MTX discontinuation or on the last follow-up for patients who were still on MTX. The dose of thalidomide was also decreased, from a mean dose of 112 mg (50-200 mg) at MTX initiation to a mean dose of 18 mg (0-50 mg) at MTX discontinuation or on the last follow up.

# MTX discontinuation and relapse

The median time of follow-up after MTX discontinuation was 17.5 months (range 3-25).

Among the 10 patients with GR or PR, 7 patients (70%) completed their treatment and 3 (30%) are still under MTX treatment. MTX was stopped because of side effects despite efficacy in 3 patients, and in 4 patients (3 women, 1 man) who were planning to have a child after a prolonged remission. Among the 7 patients who stopped MTX after good or partial response, 3 (42%) showed relapse after a median of 5.5 months (range 3-14) (*patients 2,4 and 9)*.

Regarding therapeutic switches after relapse in GR and PR: one patient was treated with a new GCs course, one patient with apremilast and one patient with certolizumab pegol.

Regarding TNF alpha inhibitors, 2 patients received certolizumab pegol in the GR group (one following relapse after MTX discontinuation and one as an alternative to MTX in the context of pregnancy). In the NR group, one patient received infliximab in addition to MTX, and one stopped MTX and started apremilast. The long-term outcome was favourable for all of them. No adverse events were reported with those treatments.

## Safety profile and adverse events

A total of 3 (23%) patients had AEs related to MTX. One patient had a grade II cytolysis that developed in a context of hepatitis B virus chronic coinfection; laboratory monitoring showed a complete recovery after stopping MTX. One patient had a grade I gastrointestinal disorder with nausea requiring MTX discontinuation. One patient had a severe grade III SARS-CoV-2 infection. This AE was considered to be partly related to several previous comorbidities such as severe hypertension, chronic renal failure and heart failure. Regarding thalidomide, grade II neuropathy was reported in two patients. Patient characteristics are summarized in **Tables 1 and 2 and Figure 1**.

**Figure 1: Flow chart of patients in the study**

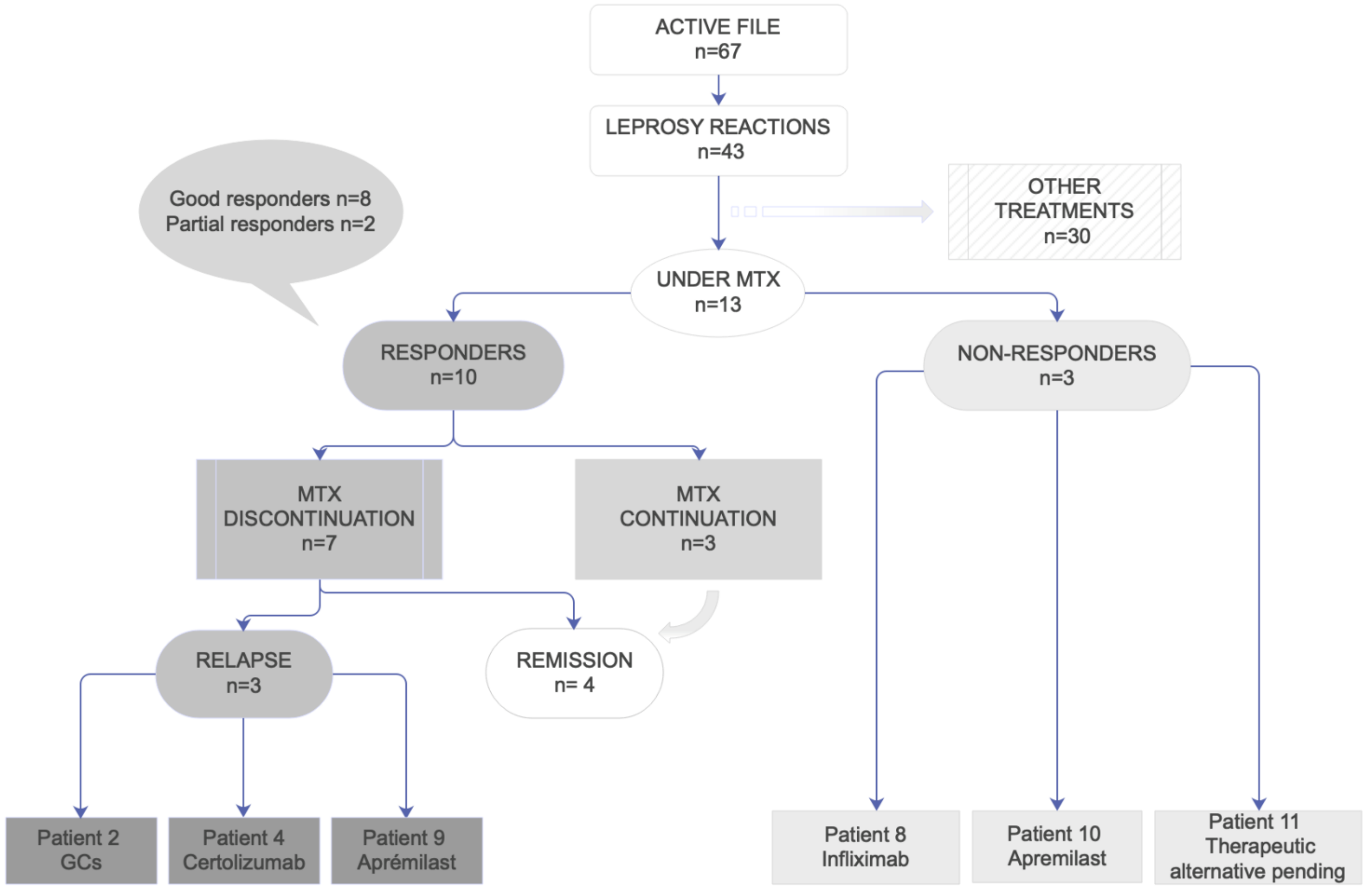

*Table 1: Characteristics of patients with leprosy (n=13)*

| | |
|---|---|
| ***Socio-demographic features*** | |
| Female / male | 5/8 |
| Age at diagnosis (years), median (range) | 29 (12-63) |
| Type of leprosy reaction:<br>- ENL<br>- RR | <br>6 (46%)<br>7 (54%) |
| ***Historical treatment of LR*** | |
| Median number of systemics before starting MTX, (range) | 2 (1-4) |
| GCs | 100% |
| Thalidomide | 9/13 (69%) |
| Others* | |
| ***Treatments continued with MTX :*** | |
| GCs | 11 (85%) |
| Mean dose of GCs at MTX initiation (range) | 46 (10-90) |
| Thalidomide | 4 (31%) |
| Mean dose of thalidomide at MTX initiation | 112.5 (50-200) |
| Pentoxifylline | 1 (9%) |
| ***MTX initiation*** | |
| Median time between 1st episode of reaction and MTX introduction (months) | 23 (1.6-131) |
| Median dose of MTX (mg/wk) | 20 (12-25) |
| MTX administration mode<br>Oral<br>Subcutaneous<br>Not reported | 4/11 (36%)<br>7/11 (64%)<br>2/13 |
| Leprosy reaction on MTX<br>RR<br>ENL | 3/13 (23%)<br>1/13 (7%)<br>2/13 (15%) |
| ***Evolution during MTX therapy*** | |
| GCs weaning under MTX | 6/11 (55%) |
| Duration of weaning GCs (months), median (range) | 6 (3-42) |
| Mean dose of GCs (mg) at MTX discontinuation or last follow up in all patients | 4 (0-20) |
| ***Evolution after stopping MTX*** | |
| Follow-up after stopping MTX (months), median (range) | 17.5 (3-25) |
| Relapse after stopping MTX among good and partial responders | 3/7 (42%) |
| Time to relapse after stopping MTX (months), median (range) | 5.5 (3-14) |

ENL: erythema nodosum leprum, MTX: methotrexate, RR: reverse reaction, mg/kg: milligrams per kilos body weight, GCs: glucocorticoids

* Pentoxifylline 4/13 (31%), colchicine 2/13 (15%)

**Table 2: Details of clinical evolution per participant**

| | Type of leprosy | Type of leprosy reaction | Previous treatment for LR | MTX administration | Minimun and maximum dose of MTX: mg/wk (mg/kg body weight) | MTX duration (months) | Time between 1st LR and MTX (months) | MTX efficacy | Side effects | MTX status (months) | Relapse after MTX discontinuation (months) | Treatment required after MTX failure or discontinuation |
|---|---|---|---|---|---|---|---|---|---|---|---|---|
| 1 | BT | RR skin and neurological (facial diplegia) | GCs | SC | 15-20 mg (0.25-0.3) | 14 | 32 | GOOD RESPONSE GCs withdrawal at 9 months of MTX introduction | None | Ongoing (14) | | |
| 2 | LL | RR skin and neurological (sensorimotor neuropathy) | GCs, thalidomide | SC | 20-25 mg (0.3-0.4) | 17 | 23.5 | GOOD RESPONSE | Gastro-intestinal disorder (nausea) | Completed | Neurological RR relapse (8): worsening of sensory neuropathy at EMG, pain hypoesthesia | GCs Restarted at 30 mg prednisone daily with gradually decreased Currently at 15 mg/day |
| 3 | LL | ENL with severity criteria: edema of the extremities, fever, MAS, episcleritis, polyarthritis | GCs | SC | 15 mg (0.23) | 7 | 21.5 | GOOD RESPONSE GCs withdrawal at 10 months of MTX introduction | Stopped to become pregnant | Completed | No relapse (12) | |

| | | | | | | | | | | | | |
|---|---|---|---|---|---|---|---|---|---|---|---|---|
| 4 | LL | ENL skin (cutaneous nodules), fever and uveitis | GCs, pentoxifylline thalidomide, colchicine | SC | 15 mg (0.2) | 17 | 76.5 | GOOD RESPONSE No GCs Thalidomide withdrawal at 17 months of MTX introduction | Stopped to become pregnant Thalidomide neuropathy | Completed | Cutaneous ENL relapse (13) | Certolizumab pegol Currently at 7 months after starting treatment: no relapse to date |
| 5 | BL | ENL with severity criteria: skin (edema of the extremities, cutaneous nodules), fever, MAS and pericarditis | GCs, thalidomide | Oral | 15-20 mg (0.25-0.3) | 33 | 1.6 | GOOD RESPONSE GCs withdrawal at 23 months of MTX introduction Thalidomide withdrawal at 8 months of MTX introduction | Stopped to become pregnant | Completed | No relapse (3) | Certolizumab pegol (MTX stop for desire of pregnancy and high risk of relapse (history of severe ENL with MAS) |
| 6 | LL | RR skin and neurological (sensorimotor neuropathy), uveitis and polyarthritis | GCs Intra-ocular GCs | SC | 15-20 mg (0.2-0.28) | 17 | 21.6 | GOOD RESPONSE No GCs No relapse of uveitis at 12 months of MTX introduction | None | Ongoing (17) | | |
| 7 | LL | RR skin and neurological | GCs, thalidomide, pentoxifylline | Oral | 17.5-25 mg (0.23-0.35) | 50 | 58.9 | PARTIAL RESPONSE Adding thalidomide, and thalidomide withdrawal 1.5 year after MTX introduction Pentoxifylline and GCs withdrawal 3.5 years after MTX introduction | Stopped to become pregnant | Completed | No relapse (12) | |

| | | | | | | | | | | | | |
|---|---|---|---|---|---|---|---|---|---|---|---|---|
| 8 | LL | ENL: skin (cutaneous nodules) | GCs, colchicine, thalidomide | Oral | 10-20 mg (0.22-0.45) | 9,8 | 22.1 | NON-RESPONSE Chronic ENL with skin involvement with GCs dependence at 12.5 mg prednisone | None | Ongoing (10) | | Infliximab added 9 months after MTX introduction At third infliximab infusion ENL not yet controlled |
| 9 | BL | RR skin and neurological (sensory neuropathy) and recurrent uveitis | GCs, thalidomide, pentoxifylline | SC | 20 mg (0.25) | 5 | 118.9 | GOOD RESPONSE GCs withdrawal at 4 months of MTX introduction | Grade II cytolysis (HBV co-infection) | Completed | Uveitis relapse (4) | Intra-ocular injections of GCs and introduction of apremilast |
| 10 | LL | RR skin and neurological (radial motor mononeuropathy, sensory neuropathy) | GCs, thalidomide | ND | 20-25 mg (0.23-0.3) | 8 | 17.5 | NON-RESPONSE RR with skin involvement with GCs dependence at 15 mg prednisone | None | Completed | Persistent RR skin flare-ups | Thalidomide introduction without efficacy on RR Relayed by apremilast |
| 11 | BL | RR neurological (chronic sensory neuropathy) | GCs | Oral | ND | 11 | 131.1 | NON-RESPONSE No improvement of the sensory neuropathy | None | Completed | Progressive worsening of sensory neuropathy (EMG and clinical) | The relay by pentoxifylline or an anti-TNF-α inhibitors is under discussion |
| 12 | LL | ENL skin (edema of the extremities, cutaneous nodules), polyarthritis and uveitis | GCs, thalidomide, pentoxifylline | SC | 10-20 mg (0.15 0.3) | 6 | 8.7 | GOOD RESPONSE Early discontinuation of all treatments (COVID-19) | COVID-19 | Completed | No relapse (16) | |

| | | | | | | | | | | |
|---|---|---|---|---|---|---|---|---|---|---|
| 13 | LL | ENL skin (edema of the extremities, cutaneous nodules) and neurological (sensorimotor neuropathy) | GCs, thalidomide | Oral | 10-17.5 mg (0.12-0.25) | 28 | 42.1 | PARTIAL RESPONSE<br>2 relapses: ENL skin and neurological 1 and 3 months after GCs withdrawal<br>GCs dependence at 5 mg prednisone and thalidomide | None<br>Thalidomide neuropathy | Ongoing (28) |

LL: lepromatous leprosy, BL: borderline lepromatous leprosy, BT: borderline tuberculoid leprosy, LR: leprosy reaction, ENL: erythema nodosum leprum, RR: reverse reaction, SC: subcutaneous, ND: not defined, MTX: methotrexate, GCs, glucocorticoids; HBV, hepatitis B virus, EMG, electromyography, MAS, macrophagic activation syndrome, TNF, tumor necrosis factor; IV, intravenous

# Discussion

This study is one of the largest cohorts reporting the benefit of MTX for treating LR (both RR and ENL) and allowing for GCs-sparing. In total, 77% of patients (n=10) were considered responders (GR and PR); 62% showed complete response, defined as a total regression of inflammatory symptoms (cutaneous, neurological and systemic) and no recurrence during treatment. However, the high relapse rate, 42%, after stopping treatment argues for suspensive rather than durable efficacy, which highlights the interest of prolonged MTX treatment to maintain a clinical response, limit relapse upon discontinuation, and avoid iterative courses of GCs. Moreover, MTX could be an option in refractory and/or cortico-dependent LRs and also in earlier stages. TNF-α inhibitors were used in 3 patients, with good safety profile long-term outcome: infliximab was used in addition to MTX for 1 patient and certolizumab pegol was used following relapse after stopping MTX for 2 patients.

We acknowledge a few limitations to our study. Our first limitation is the retrospective design. The second one is the heterogeneity of patient profiles. Certainly, the lack of a standardized protocol in patient management may have reduced subjectivity in the evaluation of treatment response. In the absence of a comparative group, the effect of MTX may have been overestimated, qualifying cases of spontaneous improvement as responses. Our sample size was small although it is the largest cohort in the literature.

Our data were globally concordant with the literature; however some points must be highlighted. Firstly, concerning the MTX course, the dosing and administration regimen differed. Our median MTX dose was twice as high as that in the literature (20 mg/week corresponding to 0.29 mg/ kg body weight *versus* 7.5 mg/week corresponding to 0.14 mg/kg body weight) and was mostly administered subcutaneously, while in contrast, for all the cases in the literature, MTX was given orally. Our therapeutic choice is based on several publications

that suggest better efficacy of subcutaneous than oral treatment in inflammatory diseases such as psoriasis and rheumatoid arthritis, with a median dose of 0.3 mg/kg/week usually used in inflammatory diseases (16), (30), (31).

Secondly, the GCs tapering schedule was faster in our patients than in literature case reports (median of 6 *versus* 12 months) with a higher relapse rate under MTX in our cohort (3/13 cases *versus* 2/30 in the literature). Finally, MTX was introduced earlier after a primary episode of LR in the literature cases (median of 11 *versus* 23 months after an initial diagnosis of LR in our cases) but with a similar median of 2 previous treatments. We identified 7 published studies including 30 cases in PubMed. One was a randomized study from Bangladesh comparing the efficacy and safety of MTX plus prednisolone (n=10 patients) *versus* prednisolone monotherapy (n=9) during 6 months in ENL. The others were case reports (18–25). Moreover, the median time on MDT was longer in our patients than in published cases. In our practice, we usually prolong the treatment according to the decrease in the bacillary index, which can be longer than the one to two years recommended by the WHO in order to prevent relapses and LR.

Currently, the first-line treatment of LR is based on prolonged GCs therapy resulting in high cumulative dose, thereby increasing the risk of adverse effects (32),(33). However, about 40% of individuals with RR do not show clinical improvement; especially with nerve damage, GCs seem to be ineffective (34) (35). In regards to the management of ENL, the chronic course and frequency of relapses (40% of ENL) are difficult to prevent and often justify prolonged and/or repeated courses of GCs(36).

Azathioprine (AZA) is an immune-suppressant drug used in immune mediated diseases(37). In AZALEP, a randomised double-blind study of 345 patients in India with RR, adding AZA to GCs did not show improvement of dermatological and neurological outcomes (8).

Ciclosporin has also been tested in a randomised trial in Ethiopia comparing ciclosporin and prednisone efficacy and the tolerance profile in acute RR. The 2 groups did not differ in improved clinical outcomes (skin and nerve), tolerance profile or rate of RR recurrence (85%). Moreover, patients on ciclosporin required additional prednisolone to treat recurrences.(9)

Clofazimine is widely used for treating ENL; it has no effect on acute episodes but might be effective in preventing chronic and recurrent ENL (11) (12) (13). The induced skin pigmentation is sometimes stigmatising and is one of the limiting factors in its long-term use(38). Thalidomide is another effective therapeutic option in moderate to severe ENL. A retrospective study of 102 patients with ENL in India argues for early initiation of thalidomide to achieve faster and longer remission owing to its efficacy in preventing recurrences (10). However, Thalidomide would be considered an irrelevant option in leprosy patients with nerve damage due to its neurotoxicity (10,11). Its use is also limited in young women not on contraception due to its teratogenicity (39),(40).

Other therapeutic alternatives such as pentoxifylline and apremilast (16 cases), an oral phosphodiesterase-4 inhibitor sharing a common molecular structure with thalidomide, seem to avoid recurrence and allow GCs-sparing(15,41–44)

Lastly, some case series concerning the value of biotherapies such as TNF-α inhibitors have recently been published. One review identified 4 published cases of refractory ENL effectively treated with a TNF-α inhibitor (infliximab or etanercept) (14). All patients previously received thalidomide and prednisone; 2 also received pentoxifylline and one AZA. GCs-sparing was reported in all patients under anti-TNFα, and 3 showed long-term remission after treatment discontinuation. These data are promising and must be confirmed in larger cohorts. Unfortunately, few therapies have demonstrated prolonged efficacy after treatment discontinuation. TNF-α inhibitors seem to have a long-lasting immunological action that can

modify the evolutionary trajectory of the disease, but the use of anti-TNF-α is limited by the cost of agents and the availability in developing countries.

In this case series of one of the largest cohorts of patients receiving MTX for LR, we described the potential of MTX as an effective alternative treatment, allowing for GCs-sparing with a good safety profile. Therefore, MTX could be considered as a first-line therapy combined with GCs to facilitate GCs-sparing and reduce the risk of recurrence and transition to chronic LR. A large randomized controlled trial is underway to assess the efficacy of MTX in conjunction with prednisolone in managing ENL(45).

"The authors received no specific funding for this work."

# References

1. Maymone MBC. Leprosy: Clinical aspects and diagnostic techniques. J AM ACAD DERMATOL. 2020;83(1):14.

2. Scollard DM, Adams LB, Gillis TP, Krahenbuhl JL, Truman RW, Williams DL. The continuing challenges of leprosy. Clin Microbiol Rev. avr 2006;19(2):338‑ 81.

3. Kamath S, Vaccaro SA, Rea TH, Ochoa MT. Recognizing and managing the immunologic reactions in leprosy. J Am Acad Dermatol. oct 2014;71(4):795‑ 803.

4. Lockwood DNJ, Lucas SB, Desikan KV, Ebenezer G, Suneetha S, Nicholls P. The histological diagnosis of leprosy type 1 reactions: identification of key variables and an analysis of the process of histological diagnosis. J Clin Pathol. mai 2008;61(5):595‑ 600.

5. Fonseca AB de L, Simon M do V, Cazzaniga RA, de Moura TR, de Almeida RP, Duthie MS, et al. The influence of innate and adaptative immune responses on the differential clinical outcomes of leprosy. Infect Dis Poverty. 6 févr 2017;6(1):5.

6. Walker SL, Lebas E, Doni SN, Lockwood DNJ, Lambert SM. The mortality associated with erythema nodosum leprosum in Ethiopia: a retrospective hospital-based study. PLoS Negl Trop Dis. mars 2014;8(3):e2690.

7. Chandler DJ, Hansen KS, Mahato B, Darlong J, John A, Lockwood DNJ. Household costs of leprosy reactions (ENL) in rural India. PLoS Negl Trop Dis. janv 2015;9(1):e0003431.

8. Lockwood DNJ, Darlong J, Govindharaj P, Kurian R, Sundarrao P, John AS. AZALEP a randomized controlled trial of azathioprine to treat leprosy nerve damage and Type 1 reactions in India: Main findings. :22.

9. Lambert SM, Alembo DT, Nigusse SD, Yamuah LK, Walker SL, Lockwood DNJ. A Randomized Controlled Double Blind Trial of Ciclosporin versus Prednisolone in the Management of Leprosy Patients with New Type 1 Reaction, in Ethiopia. PLoS Negl Trop Dis. avr 2016;10(4):e0004502.

10. Kaur I, Dogra S, Narang T, De D. Comparative efficacy of thalidomide and prednisolone in the treatment of moderate to severe erythema nodosum leprosum: a randomized study. Australas J Dermatol. août 2009;50(3):181‑ 5.

11. Van Veen NHJ, Lockwood DNJ, Van Brakel WH, Ramirez J, Richardus JH. Interventions for erythema nodosum leprosum. A Cochrane review. Lepr Rev. déc 2009;80(4):355‑ 72.

12. Balagon M, Saunderson PR, Gelber RH. Does clofazimine prevent erythema nodosum leprosum (ENL) in leprosy? A retrospective study, comparing the experience of multibacillary patients receiving either 12 or 24 months WHO-MDT. Lepr Rev. sept 2011;82(3):213‑ 21.

13. Maghanoy A, Balagon M, Saunderson P, Scheelbeek P. A prospective randomised, double-blind, placebo controlled trial on the effect of extended clofazimine on Erythema

Nodosum Leprosum (ENL) in multibacillary (MB) leprosy. Leprosy Review. 1 juin 2017;88(2):208‑ 2016.

14. Cogen AL, Lebas E, De Barros B, Harnisch JP, Faber WR, Lockwood DN, et al. Biologics in Leprosy: A Systematic Review and Case Report. Am J Trop Med Hyg. mai 2020;102(5):1131‑ 6.

15. Narang T, Ashraf R, Kaushik A, Dogra S. Apremilast in multibacillary leprosy patients with chronic and recurrent erythema nodosum leprosum: A prospective single centre pilot study. J Eur Acad Dermatol Venereol. 8 août 2021;

16. Swierkot J, Szechiński J. Methotrexate in rheumatoid arthritis. Pharmacol Rep. août 2006;58(4):473‑ 92.

17. Warren RB, Weatherhead SC, Smith CH, Exton LS, Mustapa MFM, Kirby B, et al. British Association of Dermatologists' guidelines for the safe and effective prescribing of methotrexate for skin disease 2016. British Journal of Dermatology. 2016;175(1):23‑ 44.

18. Biosca G, Casallo S. Methotrexate Treatment for Type 1 (Reversal) Leprosy Reactions. :3.

19. Kar BR, Babu R. Methotrexate in resistant ENL. Int J Lepr Other Mycobact Dis. déc 2004;72(4):480‑ 2.

20. Nagar R, Khare S, Sengar SS. Effectiveness of Methotrexate in prednisolone and thalidomide resistant cases of Type 2 lepra reaction: report on three cases. LEPROSY. 1 déc 2015;86(4):379‑ 82.

21. Saxena S, Khurana A, B S, Sardana K, Agarwal A, Muddebihal A, et al. Severe type 2 leprosy reaction with COVID-19 with a favourable outcome despite continued use of corticosteroids and methotrexate and a hypothesis on the possible immunological consequences. Int J Infect Dis. févr 2021;103:549‑ 51.

22. Hossain D. Management of chronic neuritis with a combination regimen of lower doses prednisolone and methotrexate: a brief report. Leprosy Review. 1 mars 2016;87(1):118‑ 21.

23. Hossain D. Using methotrexate to treat patients with ENL unresponsive to steroids and clofazimine: a report on 9 patients. Lepr Rev. mars 2013;84(1):105‑ 12.

24. Hasan Z, Mowla MR, Angkur DM, Khan MI. Efficacy and Safety of Prednisolone Monotherapy Versus Prednisolone Plus Methotrexate in Erythema Nodosum Leprosum (Type 2 Lepra Reaction). International Journal of Dermatology and Venereology. déc 2020;3(4):205‑ 10.

25. Perez-Molina JA, Arce-Garcia O, Chamorro-Tojeiro S, Norman F, Monge-Maillo B, Comeche B, et al. Use of methotrexate for leprosy reactions. Experience of a referral center and systematic review of the literature. Travel Med Infect Dis. oct 2020;37:101670.

26. WHO-MVP-EMP-IAU-2019.06-eng.pdf [Internet]. [cité 29 août 2021]. Disponible sur: https://apps.who.int/iris/bitstream/handle/10665/325771/WHO-MVP-EMP-IAU-2019.06-eng.pdf

27. Pavy S, Constantin A, Pham T, Gossec L, Maillefert JF, Cantagrel A, et al. Methotrexate therapy for rheumatoid arthritis: clinical practice guidelines based on published evidence and expert opinion. Joint Bone Spine. juill 2006;73(4):388‑ 95.

28. Liu L, Liu S, Wang C, Guan W, Zhang Y, Hu W, et al. Folate Supplementation for Methotrexate Therapy in Patients With Rheumatoid Arthritis: A Systematic Review. JCR: Journal of Clinical Rheumatology. août 2019;25(5):197.

29. Fardet L, Galicier L, Lambotte O, Marzac C, Aumont C, Chahwan D, et al. Development and validation of the HScore, a score for the diagnosis of reactive hemophagocytic syndrome. Arthritis Rheumatol. sept 2014;66(9):2613‑ 20.

30. Bianchi G, Caporali R, Todoerti M, Mattana P. Methotrexate and Rheumatoid Arthritis: Current Evidence Regarding Subcutaneous Versus Oral Routes of Administration. Adv Ther. mars 2016;33(3):369‑ 78.

31. Attwa EM, Elkot RA, Abdelshafey AS, Hafez AR. Subcutaneous methotrexate versus oral form for the treatment and prophylaxis of chronic plaque psoriasis. Dermatol Ther. sept 2019;32(5):e13051.

32. World Health Organization. Leprosy hansen disease: management of reactions and prevention of disabilities: technical guidance [Internet]. World Health Organization; 2020 [cité 13 sept 2021]. ix, 58 p. Disponible sur: https://apps.who.int/iris/handle/10665/332022

33. Rao PSSS, Sugamaran DST, Richard J, Smith WCS. Multi-centre, double blind, randomized trial of three steroid regimens in the treatment of type-1 reactions in leprosy. Lepr Rev. mars 2006;77(1):25‑ 33.

34. Van Veen NHJ, Nicholls PG, Smith WCS, Richardus JH. Corticosteroids for treating nerve damage in leprosy. Cochrane Database Syst Rev. 23 mai 2016;(5):CD005491.

35. Wagenaar I, Post E, Brandsma W, Bowers B, Alam K, Shetty V, et al. Effectiveness of 32 versus 20 weeks of prednisolone in leprosy patients with recent nerve function impairment: A randomized controlled trial. PLoS Negl Trop Dis. 4 oct 2017;11(10):e0005952.

36. Negera E, Walker SL, Girma S, Doni SN, Tsegaye D, Lambert SM, et al. Clinico-pathological features of erythema nodosum leprosum: A case-control study at ALERT hospital, Ethiopia. PLoS Negl Trop Dis. 13 oct 2017;11(10):e0006011.

37. Abu-Shakra M, Shoenfeld Y. Azathioprine therapy for patients with systemic lupus erythematosus. Lupus. 2001;10(3):152‑ 3.

38. Maia MV, Cunha M da GS, Cunha CS. Adverse effects of alternative therapy (minocycline, ofloxacin, and clofazimine) in multibacillary leprosy patients in a recognized health care unit in Manaus, Amazonas, Brazil. An Bras Dermatol. 2013;88(2):205‑ 10.

39. Chaudhry V, Cornblath DR, Corse A, Freimer M, Simmons-O'Brien E, Vogelsang G. Thalidomide-induced neuropathy. Neurology. 24 déc 2002;59(12):1872‑5.

40. Speirs AL. Thalidomide and congenital abnormalities. Lancet. 10 févr 1962;1(7224):303‑5.

41. Narang T, Kaushik A, Dogra S. Apremilast in chronic recalcitrant erythema nodosum leprosum: a report of two cases. Br J Dermatol. avr 2020;182(4):1034‑7.

42. Sales AM, de Matos HJ, Nery J a. C, Duppre NC, Sampaio EP, Sarno EN. Double-blind trial of the efficacy of pentoxifylline vs thalidomide for the treatment of type II reaction in leprosy. Braz J Med Biol Res. févr 2007;40(2):243‑8.

43. Abril-Pérez C, Palacios-Diaz RD, Navarro-Mira MÁ, Botella-Estrada R. Successful treatment of erythema nodosum leprosum with apremilast. Dermatol Ther. mars 2022;35(3):e15258.

44. Sánchez-Martínez EM, Melgosa-Ramos FJ, Moneva-Léniz LM, Gegúndez-Hernández H, Prats-Máñez A, Mateu-Puchades A. Erythema nodosum leprosum successfully treated with apremilast: more effective and safer than classic treatments? Int J Dermatol. févr 2021;60(2):e74‑6.

45. London School of Hygiene and Tropical Medicine. Methotrexate and Prednisolone Study in Erythema Nodosum Leprosum (MaPS in ENL [Internet]. clinicaltrials.gov; 2021 févr [cité 23 août 2021]. Report No.: NCT03775460. Disponible sur: https://clinicaltrials.gov/ct2/show/NCT03775460

Figure

Click here to access/download;Figure;Figure 1.tif

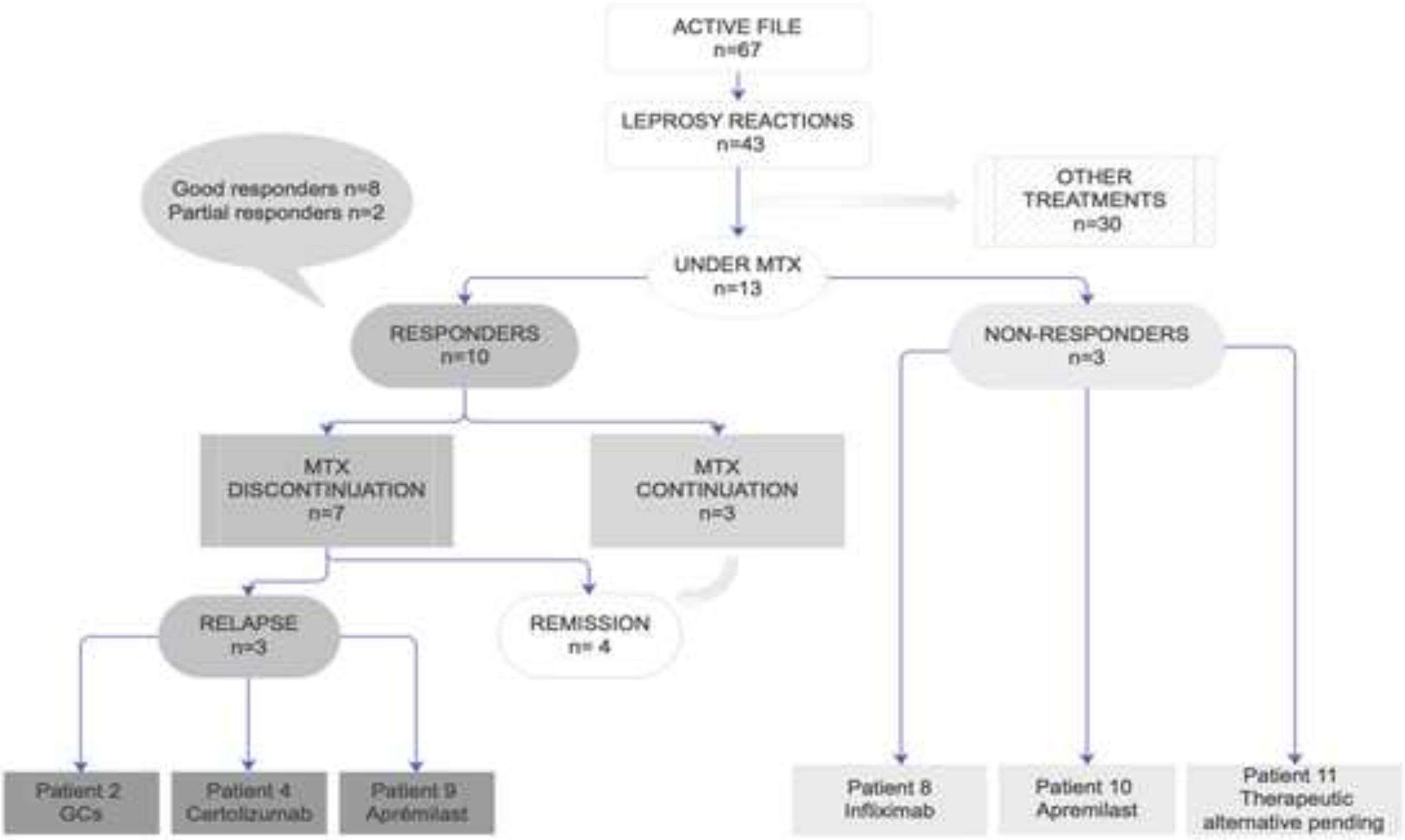

Dear Editor,

We would like to thank you and the reviewers for the careful reading of our manuscript and their comments, which we found very helpful.

Here, you will find a point-by-point response to all queries raised by the reviewers and editors. The changes made in the manuscript have been highlighted using the track-change mode. As requested, we have submitted a marked-up version and a clean version.

Reviewers' comments

***Methods***

- Reviewer #1: Small sample size

**Response: We agree with the small sample size, which reflects the difficulty of gathering multicenter cases of LR especially chronic forms that are difficult to treat. We discuss the small sample size in the limitations. (Please see page 22, line 331)**

- Reviewer #2: Slit skin smear of cases not given.

**Response: Slit skin smears were not performed systematically but skin biopsy are available for all the patients with cutaneous lesions.**

No Histopathology examination done to differentiate Type 1 from 2 reactions as it is unusual to both exist together as was in two of cases

**Response: We thank the reviewer for these important comments. We described 2 patients with both RR and ENL because they developed either an RR or an ENL during follow up. In order to clarify the manuscript, we modify and remove the RR+ENL category. We classified the 2 patients as RR or ENL depending on the predominant reaction that justified the introduction of MTX: RR was predominant and the reason of MTX introduction in these 2 cases.**

Inclusion criteria not clear

Response: **We specified inclusion criteria: leprosy patients with type 1 or type 2 LR treated at least 3 months with MTX, between June 2016 and August 2021 in France (Please see page 6, lines 119-120).**

Dose of Methotrexate - how calculated not given

Dose of Corticosteroids nor given

**Response: Median dose and range of GCs were given in the text but not in Table 1. We added GCs dosage in Table 1 to clarify the revised manuscript.**

**Dosage of MTX and GCs were collected retrospectively and the mg/kg/wk for MTX and mg/kg for GCs were calculated with each patient weight.**

***Results***

- Reviewer #1: yes results are clear and tables are sufficient quality

**Response: Thank you for your comment.**

- Reviewer #2: Outcome parameters seems subjective

  How response was attributed to methotrexate when all the patients were also in corticosteroids

and continued for more than 6 months in addition to Thalidomide and pentoxifylline in few Methotrexate is also acting drug in leprosy reactions takes 8-12 weeks to show meaningful clinical response

**Response: we thank the reviewer for this important comment.**
**We included ENL or RR patients with GCs dependance or GCs resistance. As shown in Table 2, all of them previously received GCs and 10/13 patients (77%) already received a second line of treatment without efficacy. All of them were chronic forms of LR with a median time between 1st episode of LR and MTX introduction of 23 months.**
**85 % of patients were taking GCs at MTX initiation, six of them (54.5%) achieved GCs withdrawal with a median time of 6 months. As you highlight, MTX takes 8-12 weeks to be effective. Response to MTX was assessed at MTX discontinuation or at last follow up for patient who were still on treatment with a median duration of MTX treatment of 14 months (5-44).**

- Reviewer #3: Results need some clarification

**Response: Thank you for your comment. We clarified the results in Table 1 and in the manuscript as suggested.**

***Conclusions***

- Reviewer #1: yes the study mentioned the limitations and addressed the public health

**Response: thank you very much for your appraisal**

- Reviewer #2: Not supported by the study findings

**Response: We rewrote the conclusion in order to match with the study findings and considering study limitations. Revised conclusion manuscript is as follows: "In this case series of one of the largest cohorts of patients receiving MTX for LR, we described the potential of MTX as an effective alternative treatment, allowing for GCs-sparing with a good safety profile. Therefore, MTX could be considered as a first-line therapy combined with GCs to facilitate GCs-sparing and reduce the risk of recurrence and transition to chronic LR. A large randomized controlled trial is underway to assess the efficacy of MTX in conjunction with prednisolone in managing ENL."**
**Please see page 26, lines 414-421.**

- Reviewer #3: Conclusions need to be revised in view of the study limitations

**Response: As suggested by the editors we revised the conclusion and discussion to make it more readable and in view of the study limitation. "In this case series of one of the largest cohorts of patients receiving MTX for LR, we described the potential of MTX as an effective alternative treatment, allowing for GCs-sparing with a good safety profile. Therefore, MTX could be considered as a first-line therapy combined with GCs to facilitate GCs-sparing and reduce the risk of recurrence and transition to chronic LR. A large randomized controlled trial is underway to assess the efficacy of MTX in conjunction with prednisolone in managing ENL."**
**Please see page 26, lines 414-421.**

***Editorial and Data Presentation Modifications***

- Reviewer #1: I have mentioned the minor modifications in manuscript pdf, so article can be recommended with minor revision

**Response: Thank you for your comment.**

- Reviewer #2: Terminologies and descriptions of some terms is not as used in leprosy in scientific publications. eg Multi-neuritis, Lucio vasculitis in ENL...etc

**Response: Thank you for your comment. This has been modified as suggested in the revised manuscript.**

- Thank you very much for inviting me to review this good research work on the leprosy reactions treatment. I believe that information provided by this paper is beneficial to understand the role of alternative drugs in treating and preventing LR. This is a field where information is very scarce so far.
  I have some minor comments for the authors: In the authors' summary, there is no comment on thalidomide, which is very useful, though a restricted drug, for treating type-II LR.

**Response: Thank you for your comment. This has been added in authors' summary: "Thalidomide is an effective alternative in moderate to severe ENL, but its use is limited given its numerous adverse effects." Please see page 3, lines 61-62.**

- The study period is not precise. It started in 2016, but the end is not specified.

**Response: Thank you for your comment. We started in June 2016 until August 2021. We added the study period in the revised manuscript. Please see page 6, line 120.**

- The schedule and drugs used for MDT regimens are not specified. Although treatment schemes are very standardized, there could be variations. Thus, the authors should clarify the doses and treatment lengths for MDT regimens used.

**Response: Thank you for this important comment.**
**All patients received rifampicine and clofazimine. Clofazimine was continued after completion of MDT to prevent LRs. Twelve (92%) patients received disulone, one of them was switched to ofloxacin because of a dapsone-induced anemia. One patient had a G6PD deficiency and was treated with clarithromycin. Except clofazimine, no MDT treatment was given during MTX therapy. We added these data in pages 10, lines 196-201.**

- The median time on treatment was 3.5 years (median 1.12-7.5), which is unusual. Lepromatous leprosy generally requires one to two years of treatment. The authors should discuss this extended treatment period. Have the authors considered the possibility that the MDT was prolonged in some cases of LR mistakenly believed as relapses?

**Response: We thank the reviewer for this important comment. Lepromatous leprosy generally requires one to two years of treatment according to the WHO recommendations. In our practice, we usually prolong the treatment according to the decrease in the bacillary index, which can be longer than the one to two years recommended by the WHO recommendations. In addition, several patients received clofazimine for an extended time to treat and prevent leprosy reactions. Moreover, the MDT was sometimes prolonged in some cases of doubt between leprosy reaction and leprosy relapses. We calculated median time on treatment without taking into account clofazimide. MDT was given during a median time of 2.3 years (1.12-7.5). This information was added in the revised text (Please see page 24 line 359-362).**

- GCs dosing and accumulated doses were not described. The same applies to thalidomide dosing. Dosage is essential to understanding LR's response (or lack thereof).

**Response: Thank you for your important comment. At MTX initiation, the mean dose of GCs was 46 mg/day (range 0-90) in all patients, and was reduced to a median dose of 4 mg/day (0-20) at MTX discontinuation or at the last follow-up for patients who were still on MTX for all patient (including non-responders).**
**Regarding Thalidomide, 4 patients were treated with thalidomide at MTX initiation with a mean dose of 112 mg (50-200mg) (median dose = 100 mg/day (50-200))**
**Mean dose of thalidomide at MTX discontinuation or last follow up was 18 mg (0-50) (median dose = 12.5 mg/day (0-50)).**
**Please see page 12, lines 237-243.**

- The references' format should be revised (it is not even through the bibliography)

**Response: We revised the references format in Vancouver Style according to Authors guidelines.**

- In my opinion, the study's main limitation is its retrospective design and the lack of a standardized protocol which reduces subjectivity in the evaluation of treatment response. In the absence of a comparative group, the effect of methotrexate may have been overestimated, qualifying cases of spontaneous improvement as responses. In this sense, in the subgroup analysis (GR vs PR/NR), the authors associate a better response to the early use of MTX (22 months vs 54 months), when what may have happened is that patients with refractory LR and with a lower probability of response, were treated later. The authors should discuss such limitations in the paper.

**Response: We thank the reviewer for this important comment.**
**The retrospective design and the absence of a comparative group do not allow us to conclude about the "subgroup analyses" between GR and PR/NR.**
**However, in our clinical practice we observe that treating LR earlier with MTX in association with GCs seems to facilitate GCs sparing and avoid recurrences/chronic evolution but it has to be evaluated in further prospective and controlled studies.**
**As discussed in a previous comment we included ENL or RR patients with GCs dependence or GCs resistance. As shown in Table 2, all of them previously received GCs and 10/13 patients (77%) already received a second line treatment without efficacy. All of them were chronic forms of LR with a median time between 1st episode of LR and MTX introduction of 23 months, it seems unlikely that these patients had a spontaneous improvement. We discussed such limitations in the paper (Page 22, lines 325-331).**

***<u>Regarding manuscript annotation:</u>***

- Have not mentioned the reaction severity score/ENLIST, grade of severity of ENL

**Response: We thank the reviewer for this comment. Unfortunately, we were not able to assess the reaction severity score/ENLIST retrospectively.**

- Mentioned the clinical/histological features of macrophage activation manifestation

**Response: These were mainly symptoms of macrophage activation syndrome without histological confirmation. We have removed this information because we do not have all the details about the macrophage activation syndrome.**

- For NR group, was drug resistance done with ALT?

**Response: We thank the reviewers for this important question. The search for anti-leprosy drug resistance was carried out systematically for all patients who had a positive *Mycobacterium Leprae* PCR.**

General comments: This manuscript is valuable, but needs rewriting to make it more readable. The use of tense is inconsistent. Please review and provide citations where necessary (mainly in discussion).

**Response: the manuscript has been edited by a native English speaker: Miss Britney Le.**

***<u>Specific comments editor:</u>***

- Line 86-101: Please make more succinct. Details can be added in discussion where many of these drugs are discussed again.

**Response: this paragraph has been reduced.**

- Line 106: There should be a mention that this is a retrospective study of patients treated with LR.

**Response: We added this mention (Please see page 6, line 116)**

Editor's comment

***<u>Methods:</u>***

- Line 123: Are these outcomes standard used across the world? If so, please provide citation. Otherwise state that these are outcomes that you have created for this study.

**Response: We thank the editor for this comment. As there is no standard recommendations to evaluate therapeutic response, we have used clinically valuable outcomes to evaluate MTX clinical response for this study. We added this mention in the manuscript. (Please see page 7, lines 141-142)**

- There is no mention of MTX monitoring or toxicities that were evaluated.

**Response: we added this mention (Please see page 6, lines 127-130)**

- Line 191: Sub-group analysis is usually done in RCTs, and these are always predefined. Suggest removing this sub-heading and simply describing both these groups.

**Response: As suggested by the Editor and the reviewers, we removed this sub-group analysis.**

- Line 204: please mention more details about monitoring/lack of monitoring and hepatoxocity seen or not seen.

**Response: We detailed MTX monitoring (page 7 Lines 139-140)**

- Discussion: Need significant editing and rewriting to make more readable. Use of tense is inconsistent

**Response: We thank the Editor for this comment. The manuscript has been edited by a native English speaker.**

- Please highlight that for 3/5 women, the desire to become pregnant necessitated stopping mTX

**Response: We highlighted this (please see page 12, line 251-253)**

- Line 228-229: should be in the concluding paragraph

**Response: Please see page 26, lines 417-419.**

Suggest moving lines 233-236 "We identified.." to after line 252

**Response: Please see pages 23-24 lines 355-359.**

- Line 247-250. Speculation without evidence. Please simply mention how your cohort differed from the published literature without any speculation to the reasons such as publication bias.

**Response: We corrected the revised manuscript accordingly. Please see page 23 lines 348-350.**

- Lines 253-301: Does not read well currently – please review and reorganize to ensure flow. Start with a discussion of overall outcomes of LR and standard of care with steroids and poor outcomes. Then discuss other options. For each drug, discuss its current use and outcomes from its use. For example lines 253, 259, 260 can be rewritten into a single sentence. Much of this can be consolidated from the introduction.

**Response: We rewrote this part succinctly as suggested.**

- Line 253-254: incorrect tense: GSs do not demonstrate clear benefits (please cite).

**Response: We removed this sentence to be more succinct.**

- Line 256: “real life experience” – please say “in our experience or our practice” as your experience does not negate others' experience.

**Response: We removed this sentence.**

- Line 259: please cite reference.

**Response: We added these 2 references: (page 24, line 369)**

34. *Van Veen NHJ, Nicholls PG, Smith WCS, Richardus JH. Corticosteroids for treating nerve damage in leprosy. Cochrane Database Syst Rev. 23 mai 2016;(5):CD005491.*

35. *Wagenaar I, Post E, Brandsma W, Bowers B, Alam K, Shetty V, et al. Effectiveness of 32 versus 20 weeks of prednisolone in leprosy patients with recent nerve function impairment: A randomized controlled trial. PLoS Negl Trop Dis. 4 oct 2017;11(10):e0005952.*

- Lines 261: rewrite "Azathioprine acts by inhibiting T-cell immunity. "

**Response: Please see page 24 line 374.**

- Line 265: suspensive is not a commonly used word, and its meaning here is unclear as related to 48-week treatment group. Please clarify and rewrite the entire sentence.

**Response: We rewrote this sentence succinctly (page 24, lines 376-378)**

- Line 271-Line 272: should this be in the section on glucocorticoids? Seems out of place between azathioprine and clofazimine.

**Response: We moved this sentence as suggested (Page 24, lines 371-373)**

- Line 273-274: please provide citation.

**Response: We added these citations: Page 25, lines 390.**

11. *Van Veen NHJ, Lockwood DNJ, Van Brakel WH, Ramirez J, Richardus JH. Interventions for erythema nodosum leprosum. A Cochrane review. Lepr Rev. déc 2009;80(4):355‑72*

12. *Balagon M, Saunderson PR, Gelber RH. Does clofazimine prevent erythema nodosum leprosum (ENL) in leprosy? A retrospective study, comparing the experience of multibacillary patients receiving either 12 or 24 months WHO-MDT. Lepr Rev. sept 2011;82(3):213‑21.*

13. *Maghanoy A, Balagon M, Saunderson P, Scheelbeek P. A prospective randomised, double-blind, placebo controlled trial on the effect of extended clofazimine on Erythema Nodosum Leprosum (ENL) in multibacillary (MB) leprosy. Leprosy Review. 1 juin 2017;88(2):208‑2016.*

- Line 278-280: please provide citation. Also rewrite – thalidomide is used with everyone fully knowing its teratogenicity; I doubt it is being prescribed without proper counselling and contraception. This line seems to imply that people are using it in pregnancy and teratogenicity is seen in patients receiving it.

**Response: We added these citations:**

10. *Kaur I, Dogra S, Narang T, De D. Comparative efficacy of thalidomide and prednisolone in the treatment of moderate to severe erythema nodosum leprosum: a randomized study. Australas J Dermatol. août 2009;50(3):181‑5.*

11. *Van Veen NHJ, Lockwood DNJ, Van Brakel WH, Ramirez J, Richardus JH. Interventions for erythema nodosum leprosum. A Cochrane review. Lepr Rev. déc 2009;80(4):355‑72.*

**We rewrote the sentence: Its use is also limited in young women without contraception because of its teratogenicity (page 25 line 395-397)**

- Line 281-283: could you provide more detail here? How many cases.

**Response: To date, we found 16 reported cases. We specified this and added these 4 citations. (page 25 line 398-400)**

41. *Narang T, Kaushik A, Dogra S. Apremilast in chronic recalcitrant erythema nodosum leprosum: a report of two cases. Br J Dermatol. avr 2020;182(4):1034‑7.*

42. *Sales AM, de Matos HJ, Nery J a. C, Duppre NC, Sampaio EP, Sarno EN. Double-blind trial of the efficacy of pentoxifylline vs thalidomide for the treatment of type II reaction in leprosy. Braz J Med Biol Res. févr 2007;40(2):243‑8.*

43. *Abril-Pérez C, Palacios-Diaz RD, Navarro-Mira MÁ, Botella-Estrada R. Successful treatment of erythema nodosum leprosum with apremilast. Dermatol Ther. mars 2022;35(3):e15258.*

44. *Sánchez-Martínez EM, Melgosa-Ramos FJ, Moneva-Léniz LM, Gegúndez-Hernández H, Prats-Máñez A, Mateu-Puchades A. Erythema nodosum leprosum successfully treated with apremilast: more effective and safer than classic treatments? Int J Dermatol. févr 2021;60(2):e74‑6*

- Line 292-295 should be included with summary paragraph about your study at the start of the discussion; currently seems out of place.

**Response: As suggested, we moved this at the start of the discussion. Please see page 22, lines 320-322.**

- Line 296-301: Please rewrite as a summary paragraph and include “in this case series of one of the largest cohorts of patients receiving MTX for LR, we demonstrate the potential of methotrexate as..." and include line 228

**Response: We rewrote this summary paragraph as suggested (page 26, lines 414-419).**

Having addressed all the points raised by reviewers and editorial office, we hope that the revised manuscript will meet the requirements for publication.

We would like to thank you for your time and consideration.

Best regards

Léa Jaume and Marie Jachiet, on behalf of all the authors

Click here to access/download
**Revised Article with Changes Highlighted**
Revised article with changes Highlighted R1.docx
