# Supplementary material for: Methotrexate as a corticosteroid-sparing agent in leprosy reactions: A French multicenter retrospective study
Source: PLoS Negl Trop Dis. 2023 Apr 20;17(4):e0011238. doi: 10.1371/journal.pntd.0011238 (PMC10153744; doi:10.1371/journal.pntd.0011238)
Supplement: Attachment — Submitted filename: PLOSNTD review.docx [file pntd.0011238.s002.docx]

Thank you very much for inviting me to review this good research work on the leprosy reactions treatment. I believe that information provided by this paper is beneficial to understand the role of alternative drugs in treating and preventing LR. This is a field where information is very scarce so far.

I have some minor comments for the authors:

- In the authors' summary, there is no comment on thalidomide which is very useful, though a restricted drug, for treating type-II LR.
- The study period is not precise. It started in 2016, but the end is not specified.
- The schedule and drugs used for MDT regimens are not specified. Although treatment schemes are very standardized, there could be variations. Thus, the authors should clarify the doses and treatment lengths for MDT regimens used.
- GCs dosing and accumulated doses were not described. The same applies to thalidomide dosing. Dosage is essential to understanding LR's response (or lack thereof).
- The median time on treatment was 3.5 years (median 1.12-7.5), which is unusual. Lepromatous leprosy generally requires one to two years of treatment. The authors should discuss this extended treatment period. Have the authors considered the possibility that the MDT was prolonged in some cases of LR mistakenly believed as relapses?
- The references' format should be revised (it is not even through the bibliography)

In my opinion, the study's main limitation is its retrospective design and the lack of a standardized protocol which reduces subjectivity in the evaluation of treatment response. In the absence of a comparative group, the effect of methotrexate may have been overestimated, qualifying cases of spontaneous improvement as responses. In this sense, in the subgroup analysis (GR vs PR/NR), the authors associate a better response to the early use of MTX (22 months vs 54 months), when what may have happened is that patients with refractory LR and with a lower probability of response, were treated later. The authors should discuss such limitations in the paper.
