# Supplementary material for: Methotrexate as a corticosteroid-sparing agent in leprosy reactions: A French multicenter retrospective study
Source: PLoS Negl Trop Dis. 2023 Apr 20;17(4):e0011238. doi: 10.1371/journal.pntd.0011238 (PMC10153744; doi:10.1371/journal.pntd.0011238)
Supplement: Attachment — Submitted filename: Response to Reviewers R1 07.02.2023.docx [file pntd.0011238.s003.docx]

Dear Editor,

We would like to thank you and the reviewers for the careful reading of our manuscript and their comments, which we found very helpful.

Here, you will find a point-by-point response to all queries raised by the reviewers and editors. The changes made in the manuscript have been highlighted using the track-change mode. As requested, we have submitted a marked-up version and a clean version.

Reviewers’ comments

***Methods***

- Reviewer #1: Small sample size

**Response: We agree with the small sample size, which reflects the difficulty of gathering multicenter cases of LR especially chronic forms that are difficult to treat. We discuss the small sample size in the limitations. (Please see page 22, line 331)**

- Reviewer #2: Slit skin smear of cases not given.

**Response: Slit skin smears were not performed systematically but skin biopsy are available for all the patients with cutaneous lesions.**

No Histopathology examination done to differentiate Type 1 from 2 reactions as it is unusual to both exist together as was in two of cases

**Response: We thank the reviewer for these important comments. We described 2 patients with both RR and ENL because they developed either an RR or an ENL during follow up. In order to clarify the manuscript, we modify and remove the RR+ENL category. We classified the 2 patients as RR or ENL depending on the predominant reaction that justified the introduction of MTX: RR was predominant and the reason of MTX introduction in these 2 cases.**

Inclusion criteria not clear

Response: **We specified inclusion criteria: leprosy patients with type 1 or type 2 LR treated at least 3 months with MTX, between June 2016 and August 2021 in France (Please see page 6, lines 119-120).**

Dose of Methotrexate - how calculated not given
Dose of Corticosteroids nor given

**Response: Median dose and range of GCs were given in the text but not in Table 1. We added GCs dosage in Table 1 to clarify the revised manuscript.**

**Dosage of MTX and GCs were collected retrospectively and the mg/kg/wk for MTX and mg/kg for GCs were calculated with each patient weight.**

***Results***

- Reviewer #1: yes results are clear and tables are sufficient quality

**Response: Thank you for your comment.**

- Reviewer #2: Outcome parameters seems subjective
  How response was attributed to methotrexate when all the patients were also in corticosteroids and continued for more than 6 months in addition to Thalidomide and pentoxifylline in few
  Methotrexate is also acting drug in leprosy reactions takes 8-12 weeks to show meaningful clinical response

**Response:** **we thank the reviewer for this important comment.**

**We included ENL or RR patients with GCs dependance or GCs resistance. As shown in Table 2, all of them previously received GCs and 10/13 patients (77%) already received a second line of treatment without efficacy. All of them were chronic forms of LR with a median** **time between 1st episode of LR and MTX introduction of 23 months.**

**85 % of patients were taking GCs at MTX initiation, six of them (54.5%) achieved GCs withdrawal with a median time of 6 months. As you highlight, MTX takes 8-12 weeks to be effective. Response to MTX was assessed at MTX discontinuation or at last follow up for patient who were still on treatment with a median duration of MTX treatment of 14 months (5-44).**

- Reviewer #3: Results need some clarification

**Response: Thank you for your comment. We clarified the results in Table 1 and in the manuscript as suggested.**

***Conclusions***

- Reviewer #1: yes the study mentioned the limitations and addressed the public health

**Response: thank you very much for your appraisal**

- Reviewer #2: Not supported by the study findings

**Response: We rewrote the conclusion in order to match with the study findings and considering study limitations. Revised conclusion manuscript is as follows: “In this case series of one of the largest cohorts of patients receiving MTX for LR, we described the potential of MTX as an effective alternative treatment, allowing for GCs-sparing with a good safety profile. Therefore, MTX could be considered as a first-line therapy combined with GCs to facilitate GCs-sparing and reduce the risk of recurrence and transition to chronic LR. A large randomized controlled trial is underway to assess the efficacy of MTX in conjunction with prednisolone in managing ENL.”**

**Please see page 26, lines 414-421.**

- Reviewer #3: Conclusions need to be revised in view of the study limitations

**Response: As suggested by the editors we revised the conclusion and discussion to make it more readable and in view of the study limitation. “In this case series of one of the largest cohorts of patients receiving MTX for LR, we described the potential of MTX as an effective alternative treatment, allowing for GCs-sparing with a good safety profile. Therefore, MTX could be considered as a first-line therapy combined with GCs to facilitate GCs-sparing and reduce the risk of recurrence and transition to chronic LR. A large randomized controlled trial is underway to assess the efficacy of MTX in conjunction with prednisolone in managing ENL.”**

**Please see page 26, lines 414-421.**

***Editorial and Data Presentation Modifications***

- Reviewer #1: I have mentioned the minor modifications in manuscript pdf, so article can be recommended with minor revision

**Response: Thank you for your comment.**

- Reviewer #2: Terminologies and descriptions of some terms is not as used in leprosy in scientific publications. eg Multi-neuritis, Lucio vasculitis in ENL...etc

**Response: Thank you for your comment. This has been modified as suggested in the revised manuscript.**

- Thank you very much for inviting me to review this good research work on the leprosy reactions treatment. I believe that information provided by this paper is beneficial to understand the role of alternative drugs in treating and preventing LR. This is a field where information is very scarce so far.

I have some minor comments for the authors: In the authors' summary, there is no comment on thalidomide, which is very useful, though a restricted drug, for treating type-II LR.

**Response: Thank you for your comment. This has been added in authors’ summary: “Thalidomide is an effective alternative in moderate to severe ENL, but its use is limited given its numerous adverse effects.” Please see page 3, lines 61-62.**

- The study period is not precise. It started in 2016, but the end is not specified.

**Response: Thank you for your comment. We started in June 2016 until August 2021. We added the study period in the revised manuscript. Please see page 6, line 120.**

- The schedule and drugs used for MDT regimens are not specified. Although treatment schemes are very standardized, there could be variations. Thus, the authors should clarify the doses and treatment lengths for MDT regimens used.

**Response: Thank you for this important comment.**

**All patients received rifampicine and clofazimine. Clofazimine was continued after completion of MDT to prevent LRs. Twelve (92%) patients received disulone, one of them was switched to ofloxacin because of a dapsone-induced anemia. One patient had a G6PD deficiency and was treated with clarithromycin. Except clofazimine, no MDT treatment was given during MTX therapy. We added these data in pages 10, lines 196-201.**

- The median time on treatment was 3.5 years (median 1.12-7.5), which is unusual. Lepromatous leprosy generally requires one to two years of treatment. The authors should discuss this extended treatment period. Have the authors considered the possibility that the MDT was prolonged in some cases of LR mistakenly believed as relapses?

**Response: We thank the reviewer for this important comment. Lepromatous leprosy generally requires one to two years of treatment according to the WHO recommendations. In our practice, we usually prolong the treatment according to the decrease in the bacillary index, which can be longer than the one to two years recommended by the WHO recommendations. In addition, several patients received clofazimine for an extended time to treat and prevent leprosy reactions. Moreover, the MDT was sometimes prolonged in some cases of doubt between leprosy reaction and leprosy relapses. We calculated median time on treatment without taking into account clofazimide. MDT was given during a median time of 2.3 years (1.12-7.5). This information was added in the revised text (Please see page 24 line 359-362).**

- GCs dosing and accumulated doses were not described. The same applies to thalidomide dosing. Dosage is essential to understanding LR's response (or lack thereof).

**Response: Thank you for your important comment. At MTX initiation, the mean dose of GCs was 46 mg/day (range 0-90) in all patients, and was reduced to a median dose of 4 mg/day (0-20) at MTX discontinuation or at the last follow-up for patients who were still on MTX for all patient (including non-responders).**

**Regarding Thalidomide, 4 patients were treated with thalidomide at MTX initiation with a mean dose of 112 mg (50-200mg) (median dose = 100 mg/day (50-200))**

**Mean dose of thalidomide at MTX discontinuation or last follow up was 18 mg (0-50) (median dose = 12.5 mg/day (0-50)).**

**Please see page 12, lines 237-243.**

- The references' format should be revised (it is not even through the bibliography)

**Response: We revised the references format in Vancouver Style according to Authors guidelines.**

- In my opinion, the study's main limitation is its retrospective design and the lack of a standardized protocol which reduces subjectivity in the evaluation of treatment response. In the absence of a comparative group, the effect of methotrexate may have been overestimated, qualifying cases of spontaneous improvement as responses. In this sense, in the subgroup analysis (GR vs PR/NR), the authors associate a better response to the early use of MTX (22 months vs 54 months), when what may have happened is that patients with refractory LR and with a lower probability of response, were treated later. The authors should discuss such limitations in the paper.

**Response: We thank the reviewer for this important comment.**

**The retrospective design and the absence of a comparative group do not allow us to conclude about the “subgroup analyses” between GR and PR/NR.**

**However, in our clinical practice we observe that treating LR earlier with MTX in association with GCs seems to facilitate GCs sparing and avoid recurrences/chronic evolution but it has to be evaluated in further prospective and controlled studies.**

**As discussed in a previous comment we included ENL or RR patients with GCs dependence or GCs resistance. As shown in Table 2, all of them previously received GCs and 10/13 patients (77%) already received a second line treatment without efficacy. All of them were chronic forms of LR with a median** **time between 1st episode of LR and MTX introduction of 23 months, it seems unlikely that these patients had a spontaneous improvement. We discussed such limitations in the paper (Page 22, lines 325-331).**

***Regarding manuscript annotation:***

- Have not mentioned the reaction severity score/ENLIST, grade of severity of ENL

**Response: We thank the reviewer for this comment. Unfortunately, we were not able to assess the reaction severity score/ENLIST retrospectively.**

- Mentioned the clinical/histological features of macrophage activation manifestation

**Response: These were mainly symptoms of macrophage activation syndrome without histological confirmation. We have removed this information because we do not have all the details about the macrophage activation syndrome.**

- For NR group, was drug resistance done with ALT?

**Response: We thank the reviewers for this important question. The search for anti-leprosy drug resistance was carried out systematically for all patients who had a positive *Mycobacterium Leprae* PCR.**

General comments: This manuscript is valuable, but needs rewriting to make it more readable. The use of tense is inconsistent. Please review and provide citations where necessary (mainly in discussion).

**Response: the manuscript has been edited by a native English speaker: Miss Britney Le.**

***Specific comments editor:***

- Line 86-101: Please make more succinct. Details can be added in discussion where many of these drugs are discussed again.

**Response: this paragraph has been reduced.**

- Line 106: There should be a mention that this is a retrospective study of patients treated with LR.

**Response:** **We added this mention (Please see page 6, line 116)**

Editor’s comment

***Methods:***

- Line 123: Are these outcomes standard used across the world? If so, please provide citation. Otherwise state that these are outcomes that you have created for this study.

**Response: We thank the editor for this comment. As there is no standard recommendations to evaluate therapeutic response, we have used clinically valuable outcomes to evaluate MTX clinical response for this study. We added this mention in the manuscript. (Please see page 7, lines 141-142)**

- There is no mention of MTX monitoring or toxicities that were evaluated.

**Response: we added this mention (Please see page 6, lines 127-130)**

- Line 191: Sub-group analysis is usually done in RCTs, and these are always predefined. Suggest removing this sub-heading and simply describing both these groups.

**Response: As suggested by the Editor and the reviewers, we removed this sub-group analysis.**

- Line 204: please mention more details about monitoring/lack of monitoring and hepatoxocity seen or not seen.

**Response: We detailed MTX monitoring (page 7 Lines 139-140)**

- Discussion: Need significant editing and rewriting to make more readable. Use of tense is inconsistent

**Response: We thank the Editor for this comment. The manuscript has been edited by a native English speaker.**

- Please highlight that for 3/5 women, the desire to become pregnant necessitated stopping mTX

**Response: We highlighted this (please see page 12, line 251-253)**

- Line 228-229: should be in the concluding paragraph

**Response: Please see page 26, lines 417-419.**

Suggest moving lines 233-236 “We identified..” to after line 252

**Response:** **Please see pages 23-24 lines 355-359.**

- Line 247-250. Speculation without evidence. Please simply mention how your cohort differed from the published literature without any speculation to the reasons such as publication bias.

**Response: We corrected the revised manuscript accordingly. Please see page 23 lines 348-350.**

- Lines 253-301: Does not read well currently – please review and reorganize to ensure flow. Start with a discussion of overall outcomes of LR and standard of care with steroids and poor outcomes. Then discuss other options. For each drug, discuss its current use and outcomes from its use. For example lines 253, 259, 260 can be rewritten into a single sentence. Much of this can be consolidated from the introduction.

**Response: We rewrote this part succinctly as suggested.**

- Line 253-254: incorrect tense: GSs do not demonstrate clear benefits (please cite).

**Response: We removed this sentence to be more succinct.**

- Line 256: “real life experience” – please say “in our experience or our practice” as your experience does not negate others' experience.

**Response: We removed this sentence.**

- Line 259: please cite reference.

**Response: We added these 2 references: (page 24, line 369)**

*34.	Van Veen NHJ, Nicholls PG, Smith WCS, Richardus JH. Corticosteroids for treating nerve damage in leprosy. Cochrane Database Syst Rev. 23 mai 2016;(5):CD005491.*

*35.	Wagenaar I, Post E, Brandsma W, Bowers B, Alam K, Shetty V, et al. Effectiveness of 32 versus 20 weeks of prednisolone in leprosy patients with recent nerve function impairment: A randomized controlled trial. PLoS Negl Trop Dis. 4 oct 2017;11(10):e0005952.*

- Lines 261: rewrite "Azathioprine acts by inhibiting T-cell immunity. "

**Response: Please see page 24 line 374.**

- Line 265: suspensive is not a commonly used word, and its meaning here is unclear as related to 48-week treatment group. Please clarify and rewrite the entire sentence.

**Response: We rewrote this sentence succinctly (page 24, lines 376-378)**

- Line 271-Line 272: should this be in the section on glucocorticoids? Seems out of place between azathioprine and clofazimine.

**Response: We moved this sentence as suggested (Page 24, lines 371-373)**

- Line 273-274: please provide citation.

**Response: We added these citations: Page 25, lines 390.**

*11.	Van Veen NHJ, Lockwood DNJ, Van Brakel WH, Ramirez J, Richardus JH. Interventions for erythema nodosum leprosum. A Cochrane review. Lepr Rev. déc 2009;80(4):355‑72*

*12.	Balagon M, Saunderson PR, Gelber RH. Does clofazimine prevent erythema nodosum leprosum (ENL) in leprosy? A retrospective study, comparing the experience of multibacillary patients receiving either 12 or 24 months WHO-MDT. Lepr Rev. sept 2011;82(3):213‑21.*

*13. Maghanoy A, Balagon M, Saunderson P, Scheelbeek P. A prospective randomised, double-blind, placebo controlled trial on the effect of extended clofazimine on Erythema Nodosum Leprosum (ENL) in multibacillary (MB) leprosy. Leprosy Review. 1 juin 2017;88(2):208‑2016.*

- Line 278-280: please provide citation. Also rewrite – thalidomide is used with everyone fully knowing its teratogenicity; I doubt it is being prescribed without proper counselling and contraception. This line seems to imply that people are using it in pregnancy and teratogenicity is seen in patients receiving it.

**Response: We added these citations:**

*10.	Kaur I, Dogra S, Narang T, De D. Comparative efficacy of thalidomide and prednisolone in the treatment of moderate to severe erythema nodosum leprosum: a randomized study. Australas J Dermatol. août 2009;50(3):181‑5.*

*11.	Van Veen NHJ, Lockwood DNJ, Van Brakel WH, Ramirez J, Richardus JH. Interventions for erythema nodosum leprosum. A Cochrane review. Lepr Rev. déc 2009;80(4):355‑72.*

**We rewrote the sentence: Its use is also limited in young women without contraception because of its teratogenicity (page 25 line 395-397)**

- Line 281-283: could you provide more detail here? How many cases.

**Response: To date, we found 16 reported cases. We specified this and added these 4 citations. (page 25 line 398-400)**

*41.	Narang T, Kaushik A, Dogra S. Apremilast in chronic recalcitrant erythema nodosum leprosum: a report of two cases. Br J Dermatol. avr 2020;182(4):1034‑7.*

*42.	Sales AM, de Matos HJ, Nery J a. C, Duppre NC, Sampaio EP, Sarno EN. Double-blind trial of the efficacy of pentoxifylline vs thalidomide for the treatment of type II reaction in leprosy. Braz J Med Biol Res. févr 2007;40(2):243‑8.*

*43.	Abril-Pérez C, Palacios-Diaz RD, Navarro-Mira MÁ, Botella-Estrada R. Successful treatment of erythema nodosum leprosum with apremilast. Dermatol Ther. mars 2022;35(3):e15258.*

*44. Sánchez-Martínez EM, Melgosa-Ramos FJ, Moneva-Léniz LM, Gegúndez-Hernández H, Prats-Máñez A, Mateu-Puchades A. Erythema nodosum leprosum successfully treated with apremilast: more effective and safer than classic treatments? Int J Dermatol. févr 2021;60(2):e74‑6*

- Line 292-295 should be included with summary paragraph about your study at the start of the discussion; currently seems out of place.

**Response: As suggested, we moved this at the start of the discussion. Please see page 22, lines 320-322.**

- Line 296-301: Please rewrite as a summary paragraph and include “in this case series of one of the largest cohorts of patients receiving MTX for LR, we demonstrate the potential of methotrexate as..." and include line 228

**Response: We rewrote this summary paragraph as suggested (page 26, lines 414-419).**

Having addressed all the points raised by reviewers and editorial office, we hope that the revised manuscript will meet the requirements for publication.

We would like to thank you for your time and consideration.

Best regards
Léa Jaume and Marie Jachiet, on behalf of all the authors
